# Vitamin B_12_ produced by *Cetobacterium somerae* improves host resistance against pathogen infection through strengthening the interactions within gut microbiota

**DOI:** 10.1186/s40168-023-01574-2

**Published:** 2023-06-15

**Authors:** Xiaozhou Qi, Yong Zhang, Yilin Zhang, Fei Luo, Kaige Song, Gaoxue Wang, Fei Ling

**Affiliations:** College of Animal Science and Technology, Northwest A&F University, Yangling, Shaanxi China

**Keywords:** Probiotics, *Cetobacterium*, Vitamin B_12_, Pathogen resistance, Gut microbiome, Co-occurrence network

## Abstract

**Background:**

Pathogen infections seriously affect host health, and the use of antibiotics increases the risk of the emergence of drug-resistant bacteria and also increases environmental and health safety risks. Probiotics have received much attention for their excellent ability to prevent pathogen infections. Particularly, explaining mechanism of action of probiotics against pathogen infections is important for more efficient and rational use of probiotics and the maintenance of host health.

**Results:**

Here, we describe the impacts of probiotic on host resistance to pathogen infections. Our findings revealed that (I) the protective effect of oral supplementation with *B. velezensis* against *Aeromonas hydrophila* infection was dependent on gut microbiota, specially the anaerobic indigenous gut microbe *Cetobacterium*; (II) *Cetobacterium* was a sensor of health, especially for fish infected with pathogenic bacteria; (III) the genome resolved the ability of *Cetobacterium somerae* CS2105-BJ to synthesize vitamin B_12_ de novo, while in vivo and in vitro metabolism assays also showed the ability of *Cetobacterium somerae* CS2105-BJ to produce vitamin B_12_; (IV) the addition of vitamin B_12_ significantly altered the gut redox status and the gut microbiome structure and function, and then improved the stability of the gut microbial ecological network, and enhanced the gut barrier tight junctions to prevent the pathogen infection.

**Conclusion:**

Collectively, this study found that the effect of probiotics in enhancing host resistance to pathogen infections depended on function of B_12_ produced by an anaerobic indigenous gut microbe, *Cetobacterium*. Furthermore, as a gut microbial regulator, B_12_ exhibited the ability to strengthen the interactions within gut microbiota and gut barrier tight junctions, thereby improving host resistance against pathogen infection.

Video Abstract

**Supplementary Information:**

The online version contains supplementary material available at 10.1186/s40168-023-01574-2.

## Background

Bacterial infections frequently spread from host to host by the tainted food or water contact. They rapidly spread among the host causing various diseases [1]. The use of antibiotics is now a common treatment for pathogen infections, but long-term use of these drugs can result in bacterial resistance as well as other health risks. In recent years, probiotics have gained widespread interest due to their excellent performance in disease prevention and alleviation. Probiotics exert their benefits through four main mechanisms: improvement of the barrier function, immunomodulation, metabolic function, and inhibition of potential pathogens [2, 3]. In addition to these, the presence of probiotics can also induce strong ecological and evolutionary forces to reshape the gut native microbial communities [4, 5]. Moreover, these changes in microbiome facilitate the treatment of various diseases and the maintenance of host health [6, 7]. To better utilize probiotics, the potential mechanisms of probiotics in regulating intestinal microbiota are therefore needed for further investigation.

Gut microbiota is a complex system that plays an important role in regulating physiology, immune, and nutrition of host [8–10]. Recent studies have demonstrated that gut microbes form an interactive ecological network to maintain host health, while the lack of a stable microbiota structure contributes to host disease [11, 12]. For example, antibiotic treatment increases the risk of gastrointestinal infections in humans, such as infection with *Clostridium difficile* [13]. Germ-free animals have considerable physiologic and immunologic variations from their counterparts raised conventionally, suggesting that microbiome plays a significant role in physiology and immunology of host. Moreover, changes in humans gut microbes have been strongly associated with diabetes, nonalcoholic fatty liver disease, obesity, and cancer [14–17]. Therefore, a thorough understanding of the factors that lead to microbiome variance in hosts is necessary to comprehend how microbiota affect host physiology and how to regulate microbiota to promote host health [18]. Diet is one of the most important external factors that might influence the composition of the gut microbiota [19]. A high-protein [20], high-fat diet [21], probiotics [22], and antibiotics [23] can all cause alterations in some bacterial groups. Recent studies have reported that antibiotic-treated and normally reared zebrafish exhibited different susceptibilities to infection by pathogenic bacteria, which were also observed in zebrafish with different gut microbiota compositions [24], suggesting that zebrafish is an animal model that can be used to study the correlation between gut microbes and host health [18, 25, 26].

Most of the gut microbiota’s contribution to host physiology is related to microbial metabolism, with bacteria contributing the most to ecosystem function in terms of relative genetic content [27, 28]. In general, microbes metabolize exogenous and endogenous substrates into nutrients for direct use by the host, and metabolites can also regulate the immune system by affecting the physiology and gene expression of host cells [29]. Additionally, the presence of different metabolic activity can help the microbiota occupy ecological niches and limit pathogen colonization at various sites through competition [30, 31]. Metabolites from specific classes of microbiota, such as short-chain fatty acids [32], bile acids [33], tryptophan [34], and B vitamins [35] not only affect host health, but also have an impact on interactions between host gut commensal bacteria. For example, large portion of vitamins produced by gut microbiota may be taken up by non-vitamin-producing gut microbes participating in symbiotic relationships, which suggested that vitamins may has the ability to reshape microbial communities [36, 37]. Vitamin B_12_ is a necessary water-soluble vitamin that is needed for maintaining neuronal health and hematopoiesis [38]. Some studies have shown that vitamin B_12_ deficiency can cause megaloblastic anemia and neurological disorders [39] and that supplementation is beneficial in several inflammatory diseases including sepsis, arthritis, Alzheimer’s disease, multiple sclerosis, and chronic fatigue syndrome [40]. Recent research suggested that vitamin B_12_ was crucial for maintaining human health in other areas, such as the structure and function of the gut microbiome [41]. Despite growing evidence that specific commensal microbes in the host gut can produce vitamin B_12_, the effect of microbially produced B_12_ on host resistance to bacterial infection is unclear.

Probiotics, including Bacillus and lactic acid bacteria, were used as a promising approach for decreasing infections [42]. *Bacillus velezensis* is an important biological control agent that is widely used in animal disease control [43]. Zebrafish, as an omnivorous freshwater fish belonging to the *Cyprinidae* family, shares homology with the human genome [44]. The zebrafish model is widely used in the researches of resistance against bacterial infection, gut microbiome, and disease prevention and cure [45–47]. Here, *Bacillus velezensis* and zebrafish were used as probiotic and animal models, respectively, to describe the effect of probiotics modulating the intestinal commensal bacteria of zebrafish on host resistance to pathogen infections. Specifically, we (I) investigated microbial changes in the gut caused by probiotics and analyze the key indigenous bacteria using 16S rRNA gene profiling sequencing, (II) investigated functional characteristics and key metabolites of this indigenous bacterium, using whole-genome sequencing combined with HPLC, and (III) investigated the mechanism of vitamin B_12_ in enhancing host resistance to pathogen infections.

## Results

### Protective effect of oral supplementation with *Bacillus velezensis* against *Aeromonas hydrophila* infection depends on gut microbiota

**Fig. 1:**
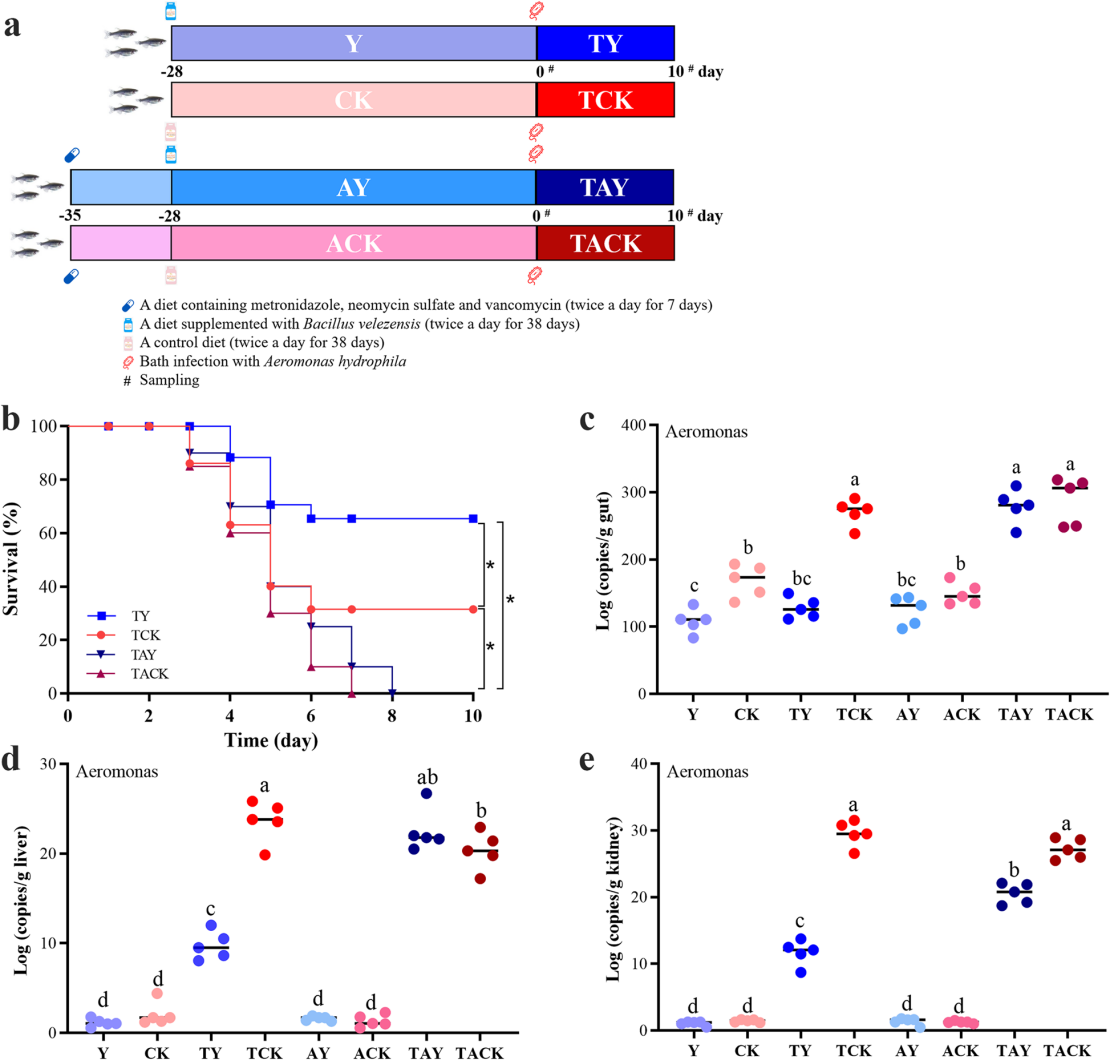
Microbiota is essential to protect fish against *A. hydrophila* infection after *B. velezensis* 1704-Y supplementation. **a** The experimental design. (i) The zebrafish were fed a basic diet supplemented with/without *B. velezensis* 1704-Y (1 × 10^7^ CFU/g diet) for 28 days (Group Y/CK), respectively, and then bath infected with *A. hydrophila* AH2006-3 J at a concentration of 1 × 10^8^ CFU/mL (Group TY/TCK); (ii) The zebrafish were fed a diet containing an antibiotic mix (120 mg/kg metronidazole, 120 mg/kg neomycin sulfate and 60 mg/kg vancomycin) for 7 days (Group AY/ACK), and then received the same treatments as (i) (Group TAY/TACK). **b** Kaplan–Meier graph of the zebrafish survival after bath infection with A. hydrophila. * indicates significant difference (*P* < 0.05) between different groups. **c–e** *Aeromonas* load (*Aero* gene copies/g of fish tissues) in fish tissues (gut, liver and kidney) sampled prior to bath infection or at 10 days post-infection. Significant differences (*P* < 0.05) between different groups are indicated with different lowercase letters above the bars

A bacterial strain 1704-Y was isolated from the zebrafish gut and identified as *Bacillus velezensis* based on morphological observation and phylogenetic analysis of *gyrB* gene (GenBank accession no. OM176702) (Fig. S1a-c) [48], and showed to be potentially safe for fish use by the hemolysis and antibiotic susceptibility tests (Fig. S1d, f; Table S2). In order to study the effects of strain 1704-Y on the prevention of pathogenic infection in fish, zebrafish were fed a basic diet supplemented with or without *Bacillus velezensis* 1704-Y for 28 days (Group Y/CK), and then bath infected with *Aeromonas hydrophila* (Group TY/TCK) (Fig. 1a). The results showed that dietary administration of BV1704-Y for 28 days significantly improved the survival rate of zebrafish bath infected with *A. hydrophila* (Group TY/TCK, *P* < 0.05, Fig. 1b). Meanwhile, a common cocktail of antibiotics was used to deplete the intestinal bacteria according to previous studies [49, 50]. Zebrafish were given mixed antibiotics (120 mg/kg metronidazole, 120 mg/kg neomycin sulfate, and 60 mg/kg vancomycin) for 1 week before feeding trial (Fig. 1a). However, no protective effect was observed when the fish were fed a diet containing an antibiotic mix for 7 days before oral administration of BV1704-Y (Fig. 1b). Moreover, dietary supplementation of BV1704-Y significantly decreased *Aeromonas* load in the fish gut, liver, and kidney after the challenge (Group TY/TCK, *P* < 0.05), whereas after administration with the antibiotic mix, the decline in *Aeromonas* load was not found in the gut and liver (Fig. 1c–e). These results suggest that a diet with the antibiotic mix removed the protective effect of oral supplementation of BV1704-Y, thereby indicating gut microbiota played a key role in the protection of fish from *A. hydrophila* infection.

### Resistance to *A. hydrophila* infection is conferred by one member of fish gut microbiota, *Cetobacterium somera*

**Fig. 2:**
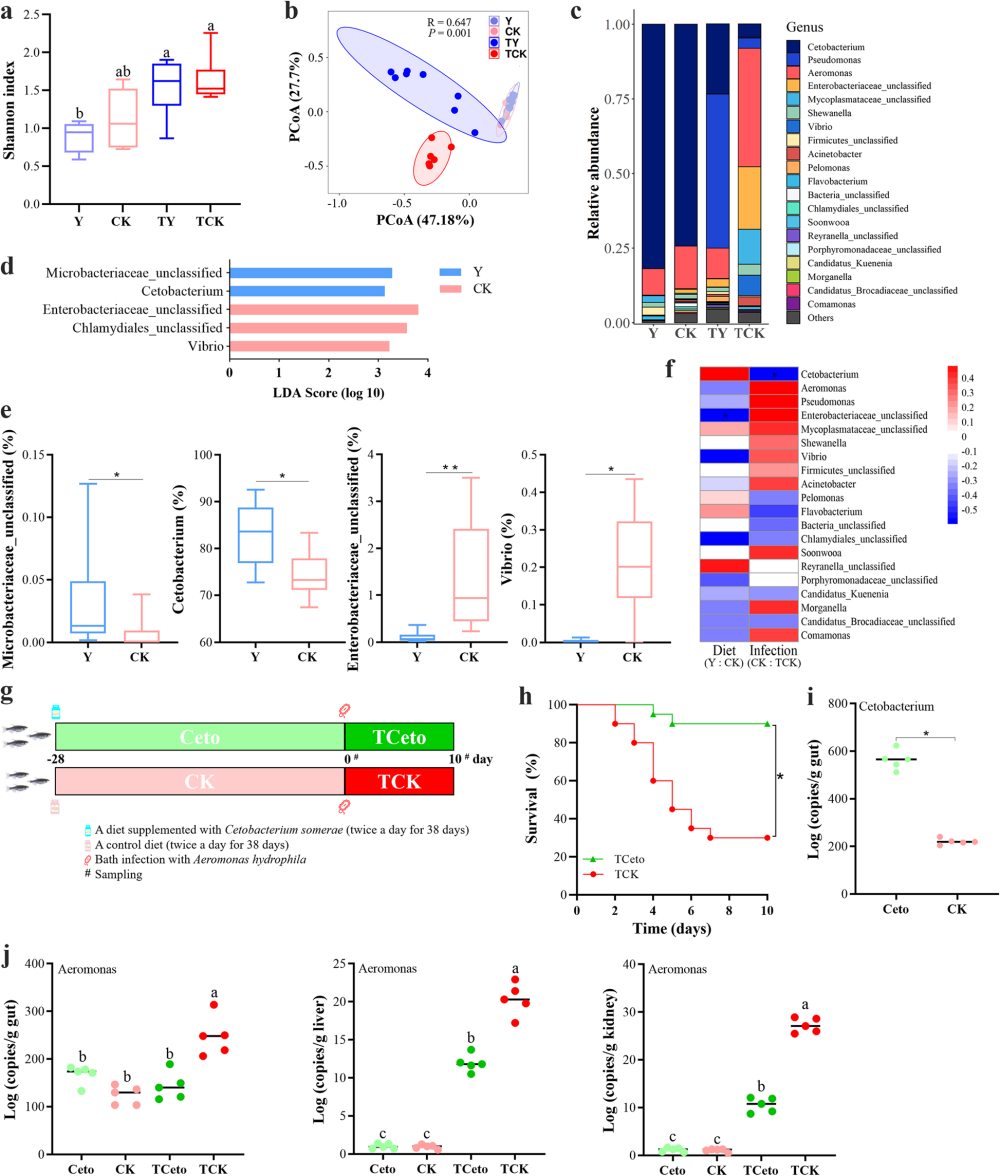
Resistance to *A. hydrophila* infection is conferred by *Cetobacterium somerae*. **a** Shannon index comparison among the different groups. The zebrafish in Group Y and CK were fed a basic diet supplemented with/without *Bacillus velezensis* 1704-Y (BV1704-Y), respectively, and then bath infected with *A. hydrophila* 2006-3 J (AH2006-3 J) at a concentration of 1 × 10^8^ CFU/mL (Group TY/TCK). **b** A principal coordinate analysis (PCoA) based on Bray–Curtis distance from the different groups (Y, CK, TY, and TCK) (ANOSIM R = 0.647, *P* = 0.001). **c** Relative abundance of the top 20 genera in the fish gut from the different groups. **d** Discriminative biomarkers identified by linear discriminant analysis effect size (LEfSe) with logarithmic LDA score > 3.0. **e** Relative abundance of selected different taxa. Data are expressed as box plot. ∗ *P* < 0.05, ∗  ∗ *P* < 0.01 by Mann–Whitney *U* test with Bonferroni-adjusted *P*-values. **f** Heat map of Pearson’s correlation coefficients between the top 20 genera and the diets (Y:CK, left) or infection status (CK:TCK, right). Dark red indicates a stronger positive correlation, dark blue indicates a stronger negative correlation, and white indicates no correlation. Black asterisk (*) means FDR-corrected *P*-value < 0.05. **g** The experimental design. The zebrafish were fed a basic diet supplemented with/without *Cetobacterium somerae* CS2105-BJ (1 × 10^7^ CFU/g diet) for 28 days (Group Ceto/CK), and then bath infected with AH2006-3 J at a concentration of 1 × 10^8^ CFU/mL (Group TCeto/TCK). **h** Kaplan–Meier graph of the zebrafish survival after bath infection with AH2006-3 J*.* **i** *Cetobacterium* load (gene copies/g of fish gut) in the gut of fish sampled prior to bath infection. **j** *Aeromonas* load (*Aero* gene copies/g of fish tissues) in fish tissues (gut, liver and kidney) sampled prior to bath infection or at 10 days post-infection. Significant differences (*P* < 0.05) between different groups are indicated with different lowercase letters

To determine how gut microbiota affected the protective effect induced by BV1704-Y, first, a 16S rRNA gene amplicon sequencing method was used to compare gut microbiota composition before and after oral administration. The results of α-diversity showed that there was no obvious difference in bacterial richness and diversity between the fish fed with and without BV1704-Y. Interestingly, *A. hydrophila* infection significantly increased the diversity of the microbiota in the fish orally administrated with BV1704-Y (Group Y/TY, *P* < 0.05), as supported by Shannon index and number of Observed_OTUs, but no significant difference was detected in these metrics (including Simpson index) of the control fish between pre- and post-infection (Fig. 2a, Fig. S2a, b). The principal coordinate analysis (PCoA) based on Bray–Curtis distances revealed that bacterial communities in the fish fed with BV1704-Y were markedly distinct from the control after bath infection with *A. hydrophila* (Group TY/TCK, ANOSIM R = 0.647, *P* = 0.001) (Fig. 2b). In addition, the composition of gut microbiota at phylum and genus levels also showed the differences in gut microbiome among different groups (Fig. 2c, Fig. S2c). The relative abundance of the most abundance phyla, Fusobacteria, was significantly increased in the fish supplemented with BV1704-Y compared to the control fish (Y vs CK, *P* = 0.0221) (Fig. S2d), while the abundance was also higher in TY group than that in TCK group (*P* = 0.0293) (Fig. S2e). A set of 9 genera were present in all samples and considered as core gut microbiome [51]. The top 5 most abundant core genera (the cumulative relative abundance was 90%) were distributed in that four dominant phyla. Among them, one genus (*Cetobacterium*) belonged to Fusobacteria, three genera (*Aeromonas*, *Enterobacteriaceae*_*unclassified*, *Shewanella*) belonged to Proteobacteria, and one genus (*Flavobacterium*) belonged to Bacteroidetes. Meanwhile, *Cetobacterium* was the genus with the highest relative abundance (67–92%) among the core microbes. Subsequently, linear discriminant analysis effect size (LEfSe) showed that two genera including *Cetobacterium* (*P* = 0.0221) and *Microbacteriaceae_unclassified* (*P* = 0.013) were enriched in Y group while another two genera including *Vibrio* (*P* = 0.0183) and *Enterobacteriaceae_unclassified* (*P* = 0.0046) were depleted compared with CK group (Fig. 2d,e). Moreover, LEfSe identified seven different bacterial genera enriched in TY group, while five other bacterial genera were depleted in TY group compared with the TCK group (Fig. S2f-r). Of note, only *Cetobacterium* was the bacterium that differed both between CK and Y groups and between TCK and TY groups. Furthermore, Pearson correlation between the top 20 most abundant genera and infection status or diet with BV1704-Y showed that bacterial taxa enriched in the Y group including *Cetobacterium* had a positive correlation with the diet containing BV1704-Y, and also had a negative correlation with infection status (Fig. 2f). Moreover, the results of the antibiotic treatment test also showed that feeding BV1704-Y did not protect zebrafish against *A. hydrophila* infection when the abundance of *Cetobacterium* in the gut was decreased (Fig. 1b, Fig. S2s). Interestingly, numerous previous studies also found that dietary supplemented with probiotics increased the abundance of *Cetobacterium* in the fish gut and protect the host from pathogenic infections [24, 52–55].

We next sought to determine if *Cetobacterium* had the ability to enhance host defenses against the pathogen infection. First, a strain of *Cetobacterium somerae* CS2105-BJ was isolated from the gut contents of healthy zebrafish, and phylogenetically characterized by 16S rRNA sequence analysis (GenBank accession no. ON248483). The characteristics of *C. somerae* CS2105-BJ was rod-shaped cells, central swelling, gram-stain-negative, and micro-aerotolerant. Subsequently, we performed an infection experiment in which the zebrafish were fed basic diets with or without *C. somerae* CS2105-BJ (CS2105-BJ) for 28 days (Group Ceto/CK), and then bath infected with *A. hydrophila* (Group TCeto/TCK) (experimental design illustrated in Fig. 2g). The results showed that the survival of the fish administrated with CS2105-BJ was significantly higher than that of the control fish after the challenge infection (*P* < 0.05, Fig. 2h). In addition, we found that dietary administration of CS2105-BJ significantly increased *Cetobacterium* load in fish gut (*P* < 0.05, Fig. 2i), and also resulted in a remarkable reduction in *Aeromonas* load in gut, liver, and kidney of the fish at 10 days post the infection with *A. hydrophila* (*P* < 0.05, Fig. 2j). Collectively, the data indicated that *C. somerae* from the zebrafish gut made a substantial contribution to protection of the fish against the bacterial infection.

### Functional annotation of complete genome unravels probiotic characteristics of *C. somerae* CS2105-BJ

**Fig. 3:**
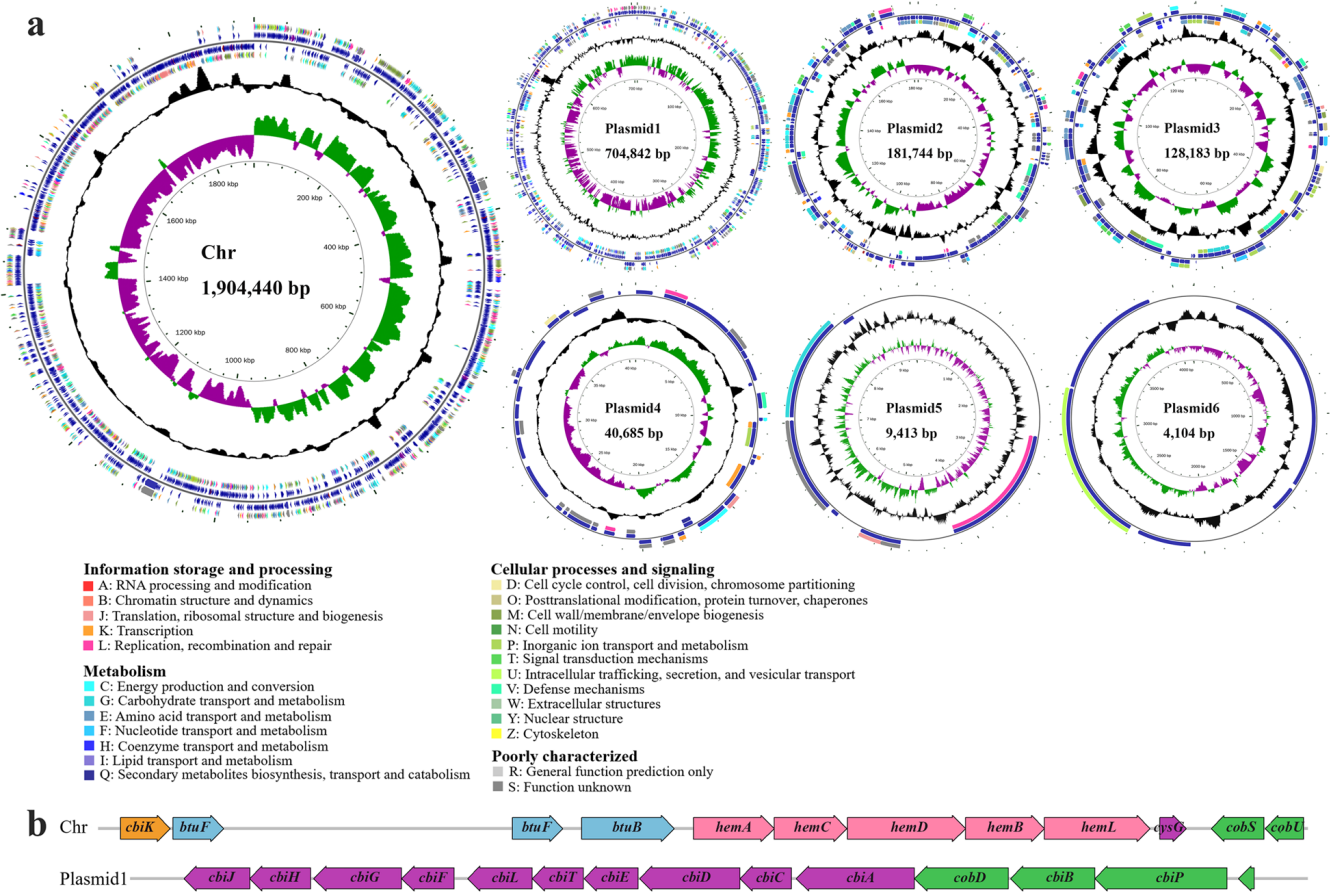
Genome analysis reveals the ability of *C. somerae CS*2105-BJ to synthesize vitamin B_12_ de novo. a Circular genomic map of CS2105-BJ chromosome and six plasmids. From the innermost to outermost circle, Circle 1 represents genome size; Circle 2 (dark purple and bottle green) represents GC skew; Circle 3 (black) shows GC plot; Circles 4 and 7 are color-coded according to the COG classification of the genes located on the forward and reverse strands, respectively. Circles 5 and 6 show the CDSs (dark blue), tRNA genes (dull red), and rRNA regions (purple). b Genomic organization of vitamin B_12_ biosynthetic genes. The pink arrows represent the genes for Uroporphyrinogen-III synthesis; the purple ones represent genes involved in the corrin ring synthesis; the orange one represents cobalt chelatase gene for insertion of cobalt ions into the corrin ring; the green ones represent genes for the attachment of the aminopropanol arm and assembly of the nucleotide loop in vitamin B_12_; the blue ones represent the genes encoding ABC transport systems for vitamin B_12_

We next performed genomic data analysis in order to get a comprehensive view of the protective potency of *C. somerae* CS2105-BJ for fish. The strain genome, de novo assembled using PacBio sequencing reads, is the first complete genome sequence available for this species and contains a single circular chromosome of 1,904,440 bp with six plasmids (plasmid 1: 704,842 bp; plasmid 2: 181,744 bp; plasmid 3: 128,183 bp; plasmid 4: 40,685 bp; plasmid 5: 9413 bp; plasmid 6: 4104 bp) (Fig. 3a). The average GC content is 29.15%. Meanwhile, there were no virulence factors found in the genome of *C. somerae* CS2105-BJ. The assembled results were also corrected with high-quality Illumina sequencing reads (Table S3). Furthermore, a total of 2732 protein-coding genes were predicted, and among them, 2371 (86.7%) genes were annotated as functional genes, and 361 (13.3%) genes were hypothetical genes. Approximately 57% of the protein-coding genes (1565 genes) were classified to 39 KEGG functional categories and 213 functional pathways (Fig. S4). Of particular concern is that the functional category of “metabolism of cofactors and vitamins” (112 genes) was the fourth most abundant categories, followed by “carbohydrate metabolism” (289 genes), “amino acid metabolism” (140 genes), and “membrane transport” (139 genes). In addition, the results of Clusters of Orthologous Groups (COG) annotations showed that the genome of CS2105-BJ has a complete set of genes required for the de novo synthesis of vitamin B_12_ (called B_12_ hereafter), also known as cyanocobalamin (Fig. 3b). It is well known that B_12_ can provide various beneficial functions to human and animal health, such as the protection of gut against gastric ulcerations, the modulation of gut microbial ecology, and the stimulation of the immune responses [56–60]. Additionally, some recent data have demonstrated that B_12_ was able to contribute to the host defense against pathogenic infection [61]. We therefore speculated that the protective effect against *A. hydrophila* infection was associated with B_12_ biosynthesis capability of *C. somerae* CS2105-BJ. We next evaluated the ability of this *C. somerae* CS2105-BJ strain to produce B_12_ in vitro and in vivo. The B_12_ production was increased with proliferation of the bacteria in vitro and reached a maximum value of 140 ng/mL (Fig. S5a). Furthermore, our results showed that oral supplementation of *C. somerae* CS2105-BJ significantly enhanced B_12_ level in the fish gut (Fig. S5b), and importantly, a strong positive correlation between B_12_ level and *Cetobacterium* load was observed (*r* = 0.9575; *P* < 0.0001) (Fig. S5c), suggesting the concentration of B_12_ in the fish gut mainly depends on *C. somerae*.

In order to further affirm the protective effect of B_12_ produced by *C. somerae*, we used excess zinc to replace cobalt ions, and thereby reduced the amount of vitamin B_12_ in the fish gut according to previous studies [62–64]. Zebrafish were randomized into four groups: control group (CK), *Cetobacterium* supplemented group (Ceto), excess zinc supplemented group (Zn), combined treatment with *Cetobacterium* and excess zinc group (Ceto + Zn). The results showed that vitamin B_12_ was significantly decreased after zinc treatment (Fig. S6a). Moreover, the concentration of B_12_ in Ceto group was significantly higher than that in CK group, and Ceto + Zn group (*P* = 0.024; *P* = 0.025, respectively) (Fig. S6a). Interestingly, after infection with *A. hydrophila*, the survival rate of zebrafish in Ceto group was also significantly higher than that in CK and Ceto + Zn groups, respectively (*P* = 0.038; *P* = 0.021) (Fig. S6b). These results suggested that vitamin B_12_ produced by gut microbes plays a key role in protecting the host from *A. hydrophila* infection. Although we did not examine other metabolites and also could not rule out the possible role of the other metabolites (may be also important), our data sufficiently support the conclusion that vitamin B_12_ is indispensable for host resistance to pathogenic infections.

### The protection effect of B_12_ against* A. hydrophila *infection is reliant on the gut microbiota

**Fig. 4:**
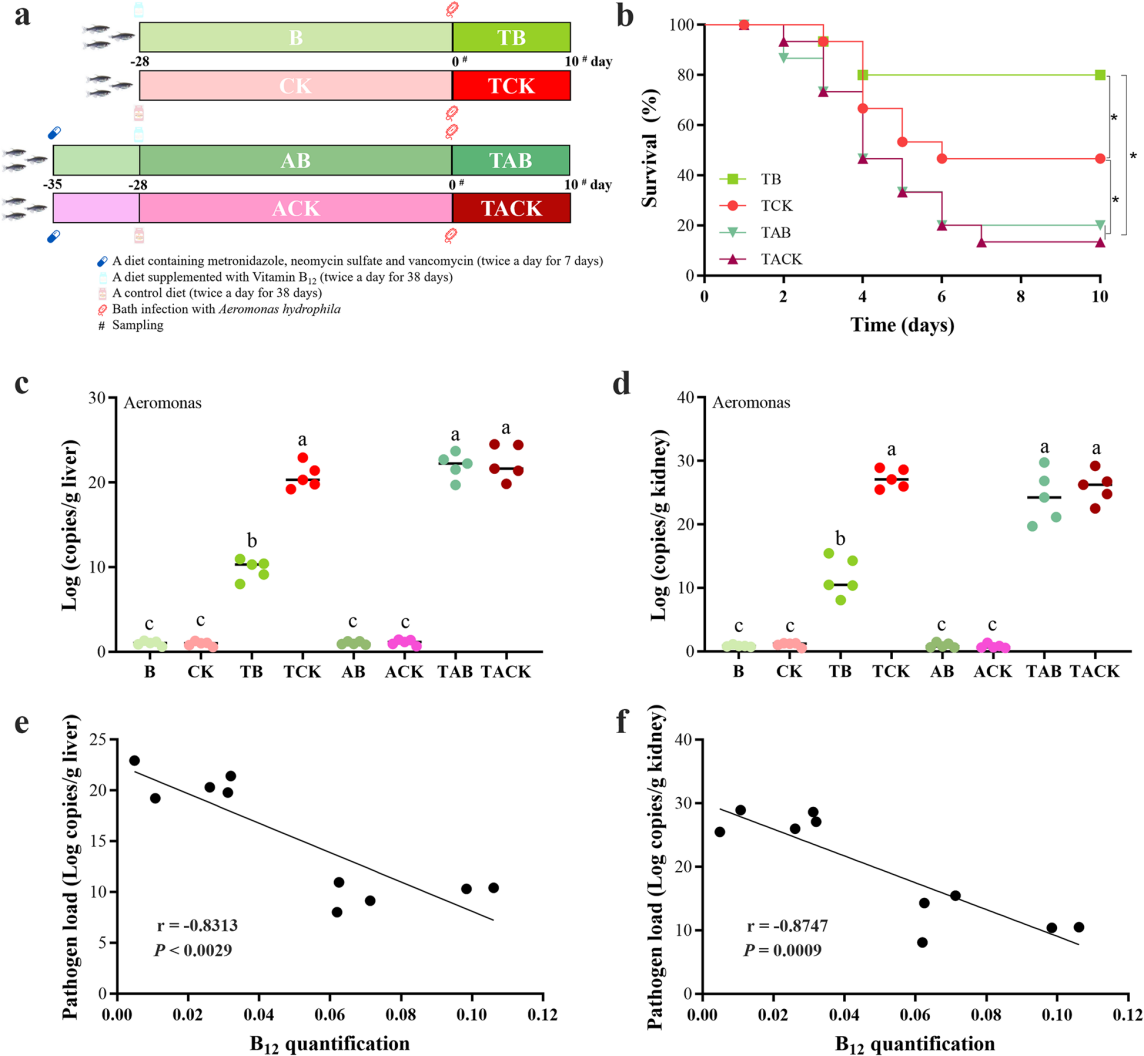
Gut microbiota are the basis of B_12_ protection against *A. hydrophila* infection in zebrafish. **a** The experimental design. (i) The zebrafish were fed a basic diet supplemented with/without vitamin B_12_ (200 μg/kg diet per day) for 28 days (Group B/CK), respectively, and then bath infected with *A. hydrophila* strain at a concentration of 1 × 10^8^ CFU/mL (Group TB/TCK). (ii) The zebrafish were fed a diet containing an antibiotic mix (120 mg/kg metronidazole, 120 mg/kg neomycin sulfate and 60 mg/kg vancomycin) for 7 days (Group AB/ACK), and then received the same treatments as (i) (Group TAB/TACK). **b** Kaplan–Meier graph of the zebrafish survival after bath infection with A. hydrophila. * indicates significant difference (*P* < 0.05) between different groups. **c, d** *Aeromonas* load (*Aero* gene copies/g of fish tissues) in fish tissues (liver and kidney) sampled prior to bath infection or at 10 days post-infection. Significant differences (*P* < 0.05) between different groups are indicated with different lowercase letters above the bars. **e**, **f** Linear correlation between the B_12_ content and pathogen load in liver and kidney, respectively. Linear correlation was performed with Pearson’s linear correlation

Then, we wonder whether B_12_ could also play a protective role against *A. hydrophila* infection. The experimental design is illustrated in Fig. 4a. Notably, dietary administration of exogenous B_12_ significantly improved the survival rate of zebrafish following bath infection with *A. hydrophila* (*P* < 0.05, Fig. 4b), and also reduced the *Aeromonas* load in liver, and kidney of the fish in TB group compared to the controls in TCK group (Fig. 4c, d). Furthermore, we observed a strong negative relationship between B_12_ level and *Aeromonas* load in liver and kidney (*r* =  − 0.9567, *P* < 0.0001; *r* =  − 0.9130, *P* = 0.0002; Fig. 4e, f). These results suggested a protective function for B_12_ in protection against *Aeromonas* infection.

More than 80% of gut microbes require exogenous B_12_, while less than 25% synthesize it themselves [65]. Moreover, B_12_ as a key metabolite in shaping the composition of the human gut microbiota has also recently gained attention [57, 66]. We postulated that the protective effect of B_12_ may be related to the gut microbiota. Previous studies in zebrafish have shown that the use of antibiotics can disrupt and clear gut microbes [67]. Before the feeding trial, we fed zebrafish with a diet containing mixture antibiotics (120 mg/kg metronidazole, 120 mg/kg neomycin sulfate and 60 mg/kg vancomycin) to deplete the commensal microbes, according to a published work (Fig. 4a) [50]. Impressively, the decline of *Aeromonas* load in the liver and kidney was not observed after the zebrafish bath infection with *A. hydrophila*, which was accompanied by an abolition of the protective effect (Fig. 4b–d). In addition, we also found that the protective effect of B_12_ on zebrafish was concentration-dependent (Fig. S7). We speculated that the small portion of high exogenous B_12_ were unabsorbed and reached the distal gut where they were available to interact with the microbiota [57, 65]. Although we cannot rule out the involvement of other mechanisms in the protection effects of B_12_, the data from these experiments support the hypothesis that B_12_ protected the host against *A. hydrophila* infection is dependent on the gut microbiota.

### Vitamin B_12_ induces alterations in gut microbiome structure and function

**Fig. 5:**
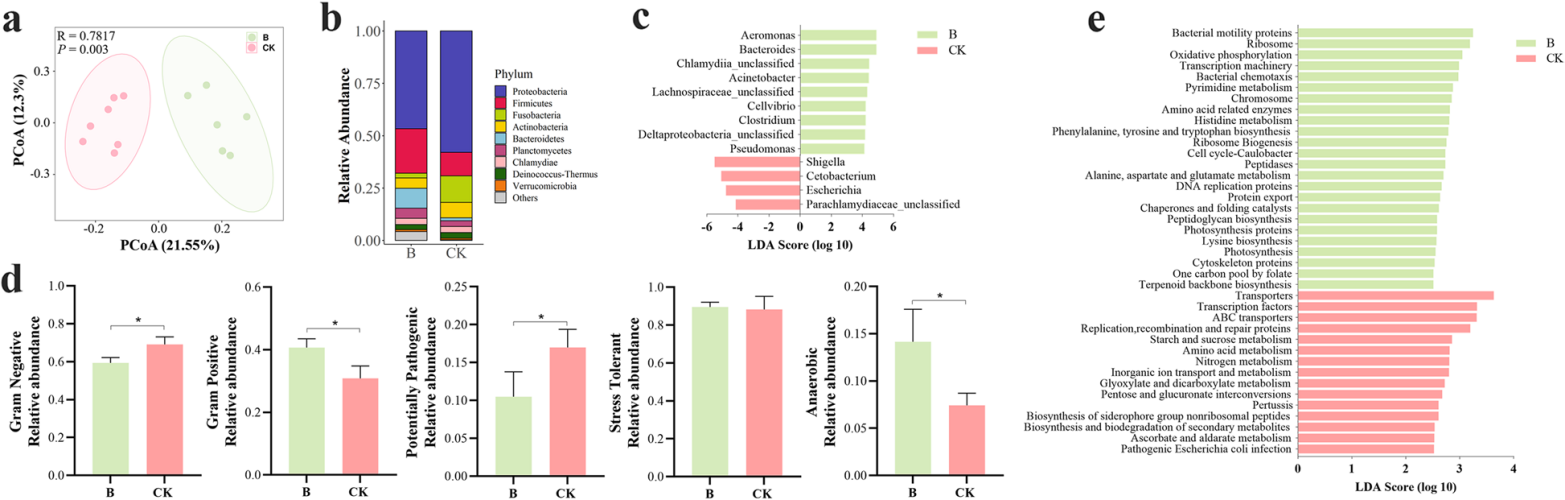
Vitamin B_12_ induces alterations in gut microbiota structure and function. **a** Principal coordinate analysis (PCoA) of Bray–Curtis distance was analyzed based on OTU level for microbiota beta diversity (ANOSIM R = 0.7817, *P* = 0.003). **b** Phylum-level taxonomic distributions of the microbial communities in gut of zebrafish fed with different diets. **c** Liner discriminant analysis effect size (LEfSe) was used to analyze the difference in microbial abundance between control and B_12_ supplemented group. The LDA value threshold was set at 4.0. **d** Bacterial community phenotypes of the gut microbiome were predicted using BugBase. Statistical significance was identified by the Wilcoxon test with false discovery rate (FDR)-corrected pairwise *P-*values. *, *P* < 0.05. **e** Functional alterations of the gut microbiome in zebrafish fed with control (CK) and B_12_-supplemented diet (B). Statistical significance was determined by using LEfSe, with a *P* value of < 0.05 (Wilcoxon test) and a linear discriminant analysis (LDA) score (log_10_) of > 2.5 being considered significant

We therefore analyzed the impact of B_12_ on the bacterial communities in the zebrafish gut. Compared to the control group (CK), the Shannon index (*P* = 0.0064) was higher in B_12_-supplemented group (B) while the Observed_OTU (*P* = 0.3343) and Chao1 index (*P* = 0.3966) was similar between the two groups (Fig. S8). To estimate the overall structure of the gut microbiota, the principal coordinate analysis (PCoA) of the variation between microbiome based on Bray–Curtis was constructed. The results showed that there was a clear separation between the two groups (ANOSIM R = 0.7817, *P* = 0.003) (Fig. 5a), suggesting that dietary administration of B_12_ significantly changed the gut microbiome community structure. Moreover, this analysis showed that the samples from TB were clustered together, suggesting that zebrafish fed with vitamin B_12_ had a more stable composition of gut microbial communities after infection with *A. hydrophila* (Fig. S9a). Similar to the results of PCoA analysis, there were significant differences in the relative abundances of several bacterial taxa between the CK and B group. The analysis at the phylum level indicated that the most abundant phyla were Proteobacteria (46.6–58.0%), Firmicutes (21.3–11.1%), and Fusobacteria (2.28–_12_.7%), followed by Actinobacteria (4.91–7.30%), Bacteroidetes (9.48–1.46%), and Planctomycetes (4.88–2.69%) (Fig. 5b). Additionally, a higher ratio of Bacteroidetes/Firmicutes (mean 0.446 versus 0.131) was found in group B compared to CK (also in group TB compared to TCK) (Fig. 5b, Fig. S9b), indicating that dietary administration of B_12_ had a huge contribution to the gut health [68, 69]. Concurrently, the heatmap of the top 50 genera also revealed a significant impact of B_12_ on gut microbiota (Fig. S10). The changes in bacterial composition between the two groups were further assessed using linear discriminant analysis effect size (LEfSe), which was used to identify the specific bacterial genera that were typical of the different treatments (Fig. 5c). The results revealed that *Shigella* and *Escherichia*, which were connected with gut microbiome dysbiosis [70, 71], were decreased in B_12_-supplemented group, while many potentially beneficial microbes, such as *Bacteroides*, *Lachnospiraceae*_uncalssified, *Cellvibrio*, and *Clostridium*, were increased.

The results of phenotype prediction according to the gut microbiome communities matched our experimental findings well (Fig. 5d). Gram-negative bacteria, which carry the most common co-pathogens [72], showed a significant decrease in abundance in group B, while gram-positive bacteria increased (*P* < 0.05), suggesting that dietary administration of B_12_ decreased potential pathogenicity. Of interest, the prediction results also displayed a significant decrease of potential pathogenicity (*P* < 0.05) in group B (Fig. 5d). In addition, the gram-negative bacteria and potential pathogenicity was significantly lower in TB group comparted to TCK group (Fig. S9d). Anaerobic bacteria were key regulators in maintaining the intestinal homeostasis [73]. The relative abundance of anaerobic bacteria drastically increased after B_12_ treatment, indicating that B_12_ might facilitate the enrichment of anaerobic microbes in gut. Moreover, the relative abundance of anaerobic bacteria in TB group was higher than that in TCK group (Fig. S9d). Moreover, the Pearson correlation coefficient revealed a strong positive correlation between the B_12_ content and the anaerobic microbiota abundance (*r* = 0.7845, *P* = 0.0015) (Fig. S11). As is widely recognized, anaerobic microbiota are greatly influenced by gut redox potential [74]. Our data indicated that the gut redox potential, a key indicator reflecting the intestinal oxygen status [73], was decreased in group B compared to control (Fig. S12a). Meanwhile, the B_12_ content correlated negatively with the redox potential in gut according to the results of Pearson correlation analysis (*r* =  − 0.8878, *P* = 0.0001) (Fig. S12b). These results suggested that dietary administration of B_12_ might reduce the redox state in the gut. Interestingly, the stress tolerance in TB group was stronger than that in TCK group (Fig. S9d), suggesting that the microbial community in the TB group was more stable. To get insights into the functional profiles of gut microbiota affected by B_12_, we performed PICRUSt analyses to predict the function of gut microbiota, and analyzed KEGG level 3 pathways with LEfSe (Fig. 5e). Compared to CK group, the sequences in group B showed enrichment of bacterial proliferation and colonization pathways involving bacterial motility proteins and bacterial chemotaxis, which might promote interactions between key constituents of the microbiota and the host [75, 76], suggesting that B_12_ mediated microbe-microbe and microbe-host interactions. After infection with *A. hydrophila*, the sequences in TB group enrichment of quorum sensing and metabolism (Fig. S9e) suggest stronger microbial interactions in the TB group [77]. Collectively, these results indicated that B_12_ supplementation significantly enhanced the diversity and community structure of the gut microbiome, and also had an impact on the redox status of the gut, improving the interaction between intestinal microbiota.

### B_12_ enhanced the complexity and stability of the gut ecological network

In the gut, various microbes interacted with each other to form a complicated ecological network to reduce the risk of disease occurrence and maintain the health of the host [12]. To identify potential interactions of the gut microbiota changed by B_12_ supplementation, we constructed co-occurrence networks using a molecular ecological network analysis pipeline (MENs) based on a random matrix theory (RMT) [78]. The samples in CK and B group each had a 0.88 threshold value applied to them [79, 80]. The networks created at the OTU level showed that all gut microbiota networks’ topologies fit the power law distribution well (R^2^ > 0.65), indicating that they possessed scale-free properties. The network total nodes, total links, average degree, average path distance, and average clustering coefficient were calculated for gut bacterial community in CK and B groups. To check the statistical significance of the created network indices, random networks were created (Table 1). Multiple network topological characteristics, including average path distance, average clustering coefficient, and connectedness, demonstrated that the gut microbial networks in B and CK were considerably different (Table 1). Compared to CK, the network in B had more nodes and links, increasing the density of connections and resulting in more complex network patterns (Fig. S13, Table 1). Meanwhile, the higher average degree, average clustering coefficient, and connectedness also reflected the increased complexity of the network in group B [81]. Collectively, these results suggested that B_12_ supplementation increased complexity of gut microbiome ecological network in zebrafish.

**Table 1:** Major topological properties of the empirical MENs of microbial communities in group CK and B and their associated random MENs

	Empirical networks								Random networks		
Group	Total nodes (*n*)	Total links (*L*)	Negative links (percentage)	Avg degree (avgK)	Avg path distance (GD)	Avg clustering coefficient (avgCC)	Connectedness (Con)	Modularity (M)	Avg path diatance (GD)	Avg clustering coefficient (avgCC)	Modularity (M)
CK	75	146	25 (17.1%)	3.893^a^	3.229^b^	0.092^c^	0.437	0.456	3.12 ± 0.113	0.091 ± 0.021	0.41 ± 0.012
B	107	323	256 (79.3%)	6.037^a^	3.014^b^	0.169^c^	0.927	0.451	2.80 ± 0.037	0.090 ± 0.013	0.33 ± 0.010

Random networks were generated by resetting all of the links of a matching empirical network with the same nodes and links. Data were generated from 100 random runs and SD indicates the standard deviation from the 100 runs

^a^Significant difference (*P* < 0.05) in average degree between CK and B based on the Student *t* test with standard deviations derived from corresponding random networks

^b^significant difference (*P* < 0.05) in average path distance between CK and B based on the Student *t* test with standard deviations derived from corresponding random networks

^c^significant difference (*P* < 0.05) in average clustering coefficients between CK and B based on the Student *t* test with standard deviations derived from corresponding random networks

**Fig. 6:**
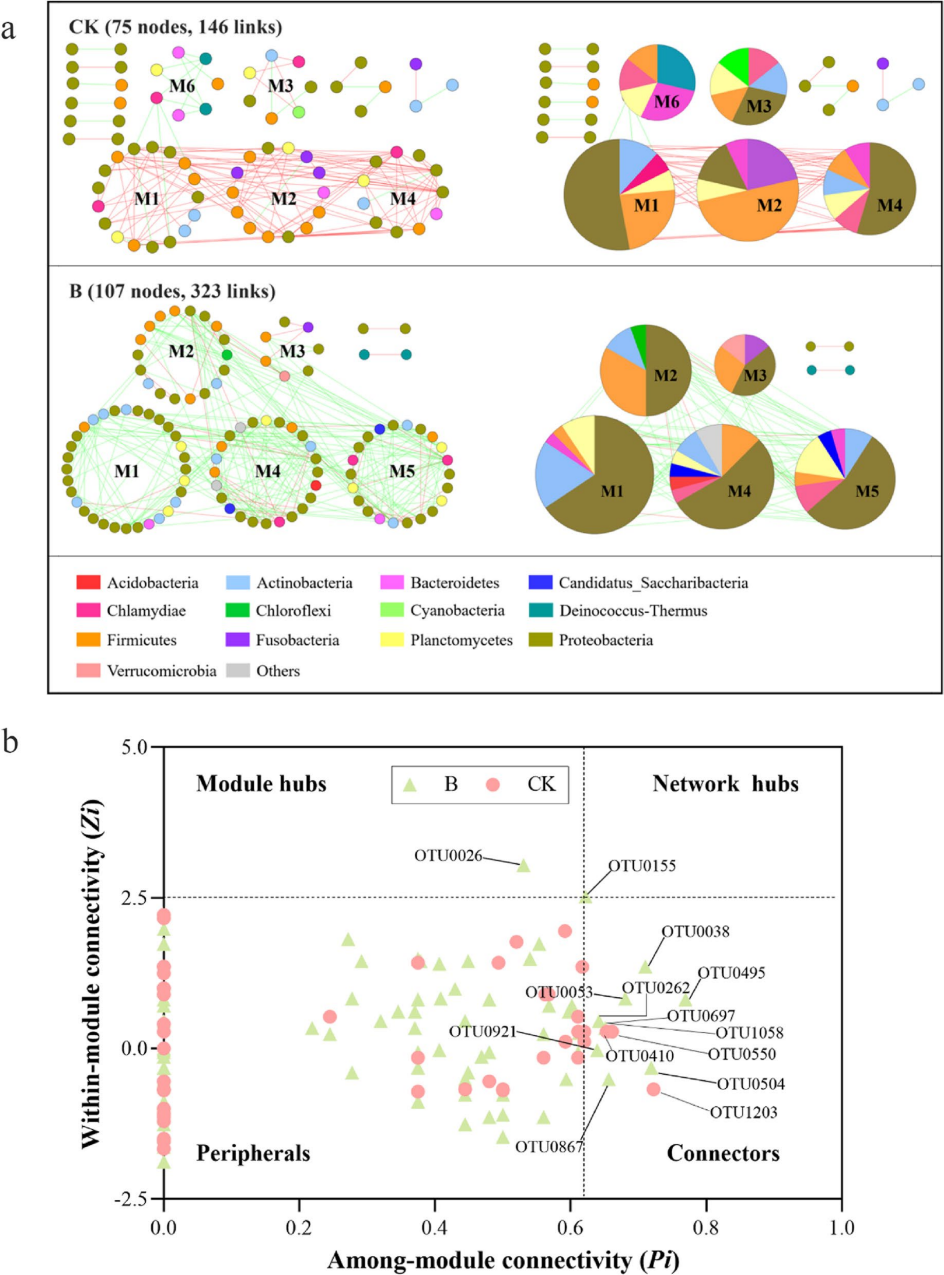
Vitamin B_12_ influence the modules and the keystone taxa in the gut ecological network. **a** Network modules in different groups. Large modules (> 5 nodes) are shown in circular layout. Major phyla are indicated by the node colors. Positive and negative correlations are indicated by red and green connections, respectively. The matching pie charts for each network in the right panel indicate the distribution of the major phyla. The module ID of each large module is indicated by M1 to M6. **b** Classification of nodes in CK and B networks to find possible keystone OTUs. Each symbol represents an OTU. Pale green symbols represent the nodes in group B. Pink symbols represent the nodes in group CK. *Zi* > 2.5 and *Pi* > 0.62 indicates network hubs; *Zi* > 2.5 and *Pi* ≤ 0.62 indicate module hubs; *Zi* ≤ 2.5 and *Pi* > 0.62 indicate connectors; and *Zi* ≤ 2.5 and *Pi* ≤ 0.62 indicate peripherals. Detailed taxonomic information for node is listed in Table S4

We concentrated on large modules (> 5 nodes) in both the CK and B networks by emphasizing the relevance of OTUs and the proportion of major phyla to find potential alterations in gut microbial interactions after B_12_ consumption. Of interest, negative correlations account for 79.3% of observed links in B network, showing that taxa generally tended to co-exclude (negative correlations, green links) rather than co-occur (positive correlations, red links), yet the opposite was true for the network in CK (Fig. 6a; Table 1), suggesting that the gut microbial ecological network in B was more stable than that in CK [82]. The network in group B had the larger modules (M1, M2, M4, and M5) that comprised many more nodes (32, 18, 24, and 22) than that in CK as a result of the network modules in group B becoming more connected (Fig. 6a). In addition, the network analyses for TCK and TB also showed that there were more negative correlations in the TB group (Fig. S14). Moreover, the network in TB group had larger modules than that in TCK group (Fig. S14), suggesting that the gut microbial ecological network in TB group was more stable than that in TCK group. Furthermore, the composition of OTUs in the modules altered significantly after B_12_ administration. Specifically, Proteobacteria and Firmicutes dominated the large modules (> 5 nodes) in CK network, while only Proteobacteria dominated the large modules (> 5 nodes) in B network (Fig. 6a). Then, the values of among-module connectivity (*Pi*) and within-module connectivity (*Zi*) of each OTU were used to identify possible keystone taxa. We classified these nodes into four groups: module hubs (*Zi* > 2.5 and *Pi* ≤ 0.62), network hubs (*Zi* > 2.5 and *Pi* > 0.62), connectors (*Zi* ≤ 2.5 and Pi > 0.62), and peripherals (*Zi* ≤ 2.5 and *Pi* ≤ 0.62) [81]. Due to their importance in the network topology, network hubs, module hubs, and connectors have been proposed as keystone taxa [81]. In this study, only one network hub and one module hub were detected in the B network. Meanwhile, compared with the CK network, the B network had more connectors (Fig. 6b), indicating that B_12_ supplementation significantly enhanced the interaction relationship between intestinal microbiota. Interestingly, the detected network hub (OTU0155) and module hub (OTU0026), as well as most of the connectors, were rare taxa, suggesting that less abundant bacteria play regulator roles in the microbial ecological network [83]. Taken together, B_12_ supplementation increased the complexity of the gut microbial ecological network and improved the interactions between gut microbes.

**Fig. 7:**
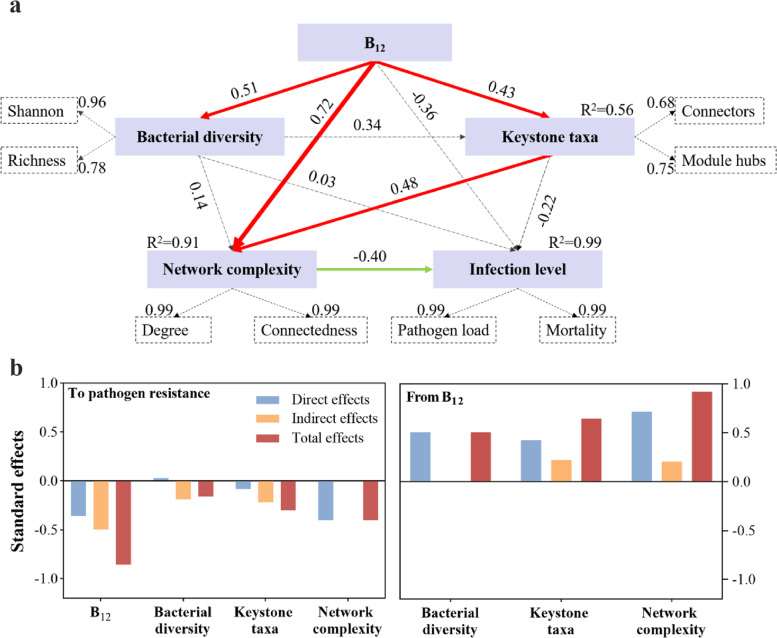
Effects of the major factors on the pathogen resistance as determined by the PLS-PM analysis. **a** PLS-PM showing the cascading relationships of different factors. An observable variable or a latent variable is represented by a box. The loading for bacterial diversity, the potential keystone taxa, the network complexity, and infection level that create the latent variables are shown in the dashed rectangles. After 1000 bootstraps, path coefficients are calculated and represented by the width of the arrow (red stands for positive relationship, green stands for negative relationship). The dashed arrow indicates a coefficient that did not differ significantly from 0 (*P* > 0.05). The GoF statistic was used to evaluate the model, and the GoF value was 0.74. **b** Standardized effects of each factor on zebrafish pathogen resistance profiles calculated from the results of partial least squares path modeling. The direct and indirect impacts are added together to form the total effects

To quantify the effects of B_12_ supplementation, microbial diversity, potential keystone taxa, and network complexity on the pathogen resistance of host, a partial least squares path model (PLS-PM) was constructed (Fig. 7a). The results indicated that B_12_ supplementation was positively correlated with bacterial diversity (0.51), network complexity (0.72), and the potential keystone taxa (0.43) but not significantly correlated with the infection level (Fig. 7). Unexpectedly, the bacterial diversity had no significant effects on the potential keystone taxa, network complexity, and infection level. Meanwhile, network complexity was also significantly impacted by the potential keystone taxa (0.48). In addition, we also found that network complexity (− 0.40) showed significant effects on the infection level, suggesting that a complex and stable network had positive effects on the pathogen resistance (Fig. 7). Overall, these results indicated that B_12_ supplementation improved host pathogen resistance mainly through influencing potential keystone species in the gut and enhancing the complexity of the gut microbial ecological network.

### B_12_ maintains normal expression of gut tight junction proteins after the pathogen infection

**Fig. 8:**
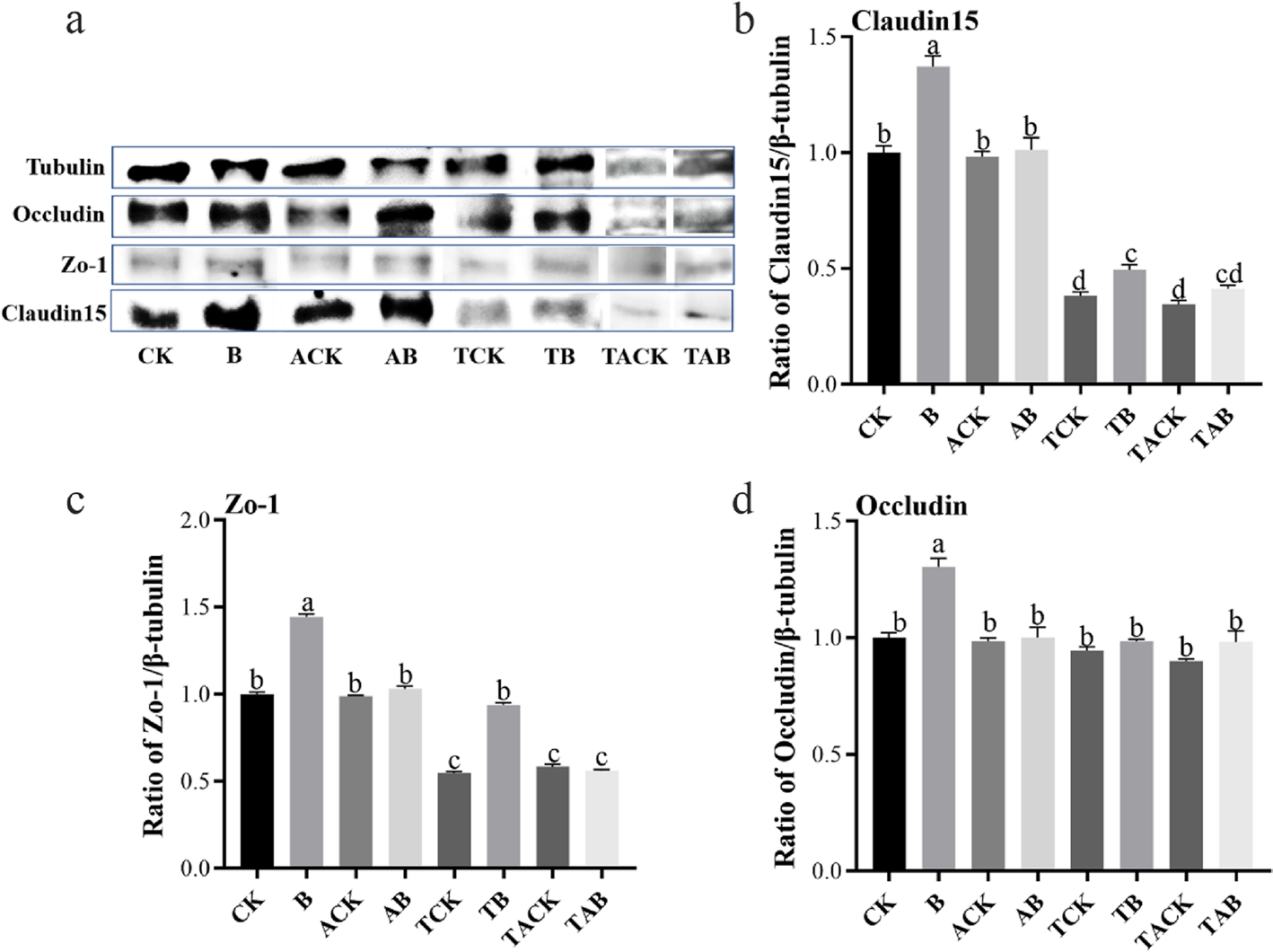
B_12_ enhances the tight junctions in the gut of zebrafish. **a** Western blots showing the expression of Zo-1, Occludin, and Claudin15 in the gut of zebrafish. **b–d** Densitometric analysis of Western blots from protein samples of the gut. Data were normalized for β-tubulin expression and expressed as fold change. Values represent means ± SD. Significant differences (*P* < 0.05) between different groups are indicated with different lowercase letters above the bars. CK: The zebrafish were fed a basic diet; B: The zebrafish were fed a basic diet supplemented with vitamin B_12_; ACK: The zebrafish treated with antibiotics for 7 days prior to administration of basic diet; AB: The zebrafish treated with antibiotics for 7 days prior to administration of B_12_; TCK: The zebrafish were fed a basic diet and then bath infected with *A. hydrophila*; TB: The zebrafish were fed a basic diet supplemented with vitamin B_12_ and then bath infected with *A. hydrophila*. TACK: The zebrafish treated with antibiotics for 7 days prior to administration of basic diet, and then fed a basic diet and then bath infected with *A. hydrophila*; TAB: The zebrafish treated with antibiotics for 7 days prior to administration of basic diet, and then fed a basic diet supplemented with vitamin B_12_ and then bath infected with *A. hydrophila*

Permeability of the gut barrier is the basis for the formation of infection outcomes in the gut by pathogens [84, 85]. Therefore, we assessed the expression of main tight junction proteins (Claudin15, Occludin, and Zo-1) of the gut by Western blot (WB). Our results showed that dietary administration of B_12_ significantly upregulated Claudin15, Occludin, and Zo-1 proteins (*P* < 0.01) (Fig. 8). In contrast, no difference in the expression of Claudin15, Occludin, and Zo-1 was observed between ACK and AB (Fig. 8), indicating that the effect of vitamin B_12_ on gut tight junction proteins disappears after antibiotics disrupt the gut microbiome. These results suggest that B_12_ influenced gut tight junction protein expression levels through the regulation of intestinal microbes. Moreover, protein levels of Claudin15 and Zo-1 after the pathogen infection were significantly decreased. Interestingly, B_12_ effectively maintained the normal expression of gut tight junction proteins in zebrafish after the infection. However, the protein levels of Claudin15, Occludin, and Zo-1 in TAB group were similar to those in TACK group, suggesting that oral administration of antibiotics significantly reduced the effect of B_12_ on the maintenance of gut barrier integrity. To further confirm these results, mRNA (*Zo-1*, *Occludin* and *Claudin15*) levels were determined by RT-PCR as well. As shown in Fig. S15, vitamin B_12_ increased *Zo-1*, *Occludin*, and *Claudin15* expression in the mRNA level (*P* < 0.05) while the infection decreased the expression of these genes. Similar to the results of western blot, RT-PCR results demonstrated that no difference in the expression of *Claudin15*, *Occludin*, and *Zo-1* was observed between ACK and AB (TACK and TAB), indicating that the effect of vitamin B_12_ on gut tight junction disappeared after the antibiotic treatment. In addition, we also analyzed the expression of intestinal tight junction proteins-related genes in zebrafish fed with *B. velezensis* and *Cetobacterium*, and found that dietary supplemented with *B. velezensis* or *Cetobacterium* significantly improved the expression of *Zo-1*, *Occludin*, and *Claudin15*, which had a similar trend with B_12_ (Fig. S16).Overall, the results of the present study suggested that B_12_-influenced gut microbiota network maintained the normal expression of gut tight junction proteins in pathogen-infected zebrafish.

## Discussion

Gut microbes are key factors in host defense against pathogen infection [86], and are incredibly important to host health [66]. In the present study, we identified a distinct mechanism by which probiotics drive intestinal commensal bacteria to produce vitamin B_12_, a microbial-derived metabolite, protecting the host from pathogenic infections. These findings highlight the relevance of the gut microbiota and associated metabolites in protecting host against the pathogen infection. Our data also support the idea of the using of *Cetobacterium* or even vitamin B_12_, as the key interventions for prevention of pathogen infection.

The characterization of gut microbiota composition is a hot topic for researchers to study the intersection of host microbiome and health [3]. It is well known that probiotics could modulate the composition and function of gut microbiota [87, 88]. We found that *Bacillus velezensis* 1704-Y supplementation could protect zebrafish against *Aeromonas hydrophila* infection by modulating the gut microbiota. Similar results were found in analogous studies in mammals, where pre-addition of *Lactobacillus* could significantly change the structure of the gut microbiome to improve the resistance to *Escherichia coli* [89]. Wang et al. found that probiotics attenuate obesity comorbidities through specific impacts on the gut microbiota in mice [90]. Moreover, studies in some animal models had demonstrated that probiotics protect host against pathogen infection through the modulation of gut microbiota [91–93]. Although the mechanisms by which probiotics regulate gut microbes still need further study, the current results all suggest that protection of the host from pathogen infections by modulating gut microbes is one of the beneficial pathways of probiotics.

Meanwhile, this study also found that *Cetobacterium*, the gut indigenous microbiota, was the key microbe to protect zebrafish against *Aeromonas hydrophila* infection after dietary supplementation of probiotic *Bacillus velezensis* 1704-Y. A growing number of studies have demonstrated that probiotic treatment can increase the relative abundance of *Cetobacterium* in the gut of freshwater fish [42, 94]. *Cetobacterium* is an anaerobic indigenous bacterium present in the gut of most freshwater fish [95]. In recent years, anaerobic indigenous gut bacteria, which played a crucial role in human health and disease, have received increasing attention from researchers. Anaerobic indigenous gut bacteria could consume dietary fiber and produce short-chain fatty acids to benefit the host. For example, *Faecalibacterium prausnitzii*, a major commensal anaerobic gut bacterium, exhibited anti-inflammatory effects on Crohn disease patients and could alleviate intestinal inflammation [96]. In addition to this, *Akkermansia muciniphila*, a mucin-degrading anaerobic bacterium, had been proved that could produce butyric acid to provide energy for gut epithelial cells and maintain the gut barrier and health [97, 98]. These suggested that gut indigenous anaerobic bacteria and their metabolites played a non-negligible role in maintaining the health of the host. We also found that *Cetobacterium* was a dominant member of gut microbiota of healthy fish, while its levels were significantly reduced in the gut of infected fish, suggesting that *Cetobacterium* was a sensor of health, especially for fish infected with pathogenic bacteria. A recent research demonstrated that *Aeromonas veronii* infection induced a significant decrease in the relative abundance of *Cetobacterium* in the gut of Yangtze finless porpoise [53]. Similarly, Ofek et al. also proved that diseased tilapia had a lower relative abundance of *Cetobacterium* in the gut compared to healthy tilapia [99]. In this study, we also found that *Aeromonas* infection significantly decreased the abundance of *Cetobacterium* in the gut. Meanwhile, both correlation analysis and antibiotic treatment tests also showed that reducing the level of *Cetobacterium* in the gut increased the susceptibility of zebrafish to pathogenic bacteria. Based on the results of previous studies and the results of this experiment, we made a reasonable hypothesis that *Cetobacterium* is a key factor maintaining the fish health and providing the protection against some pathogenic bacteria. Moreover, genomic analysis showed that *Cetobacterium* has all the genes needed to synthesize vitamin B_12_ de novo, and HPLC results from in vitro metabolism tests showed that *Cetobacterium* could produce vitamin B_12_. Approximately 80% of gut microbes appear to require vitamin B_12_, while less than 25% of gut microbes can synthesize vitamin B_12_ [65], indicating that vitamin B_12_ was an essential factor in maintaining normal life activities of bacteria. Currently, only *Fusobacteria*, *Veillonella*, *Klebsiella*, *Pseudomonas*, *Lactobacilli*, and *Bifidobacteria* had the ability to produce vitamin B_12_ in the gut [100]. Moreover, recent studies had proved that *Akkermansia muciniphila* might also had the ability to synthesize vitamin B_12_ [101]. Furthermore, *Cetobacterium* has the ability to produce vitamin B_12_ allowing it to impact these bacteria to interact with their hosts and other members of the gut microbiota, which further deepened our understanding of how this important anaerobic indigenous gut bacterium affected fish health. The data herein of microbiota and metabolism analysis suggested that *Cetobacterium* was a key anaerobic indigenous gut bacterium for maintaining host health in freshwater fish.

Here, we discovered that vitamin B_12_ has a novel function as a regulator of host gut microbial interactions, which helps to strengthen interactions within the gut microbiota and improves host resistance to pathogen infections. We found that the protective effect of B_12_ on zebrafish was concentration-dependent. We speculated that oral high-dose B_12_ supplements were largely unabsorbed and reached the distal gut where they were available to interact with the microbiota. Unlike other water-soluble vitamins, which are largely absorbed and enter the circulation, vitamin B_12_ absorption in the ileum becomes saturated around 2 μg/meal [102]. There are many studies focused their attention on studying the relationship between B_12_ receptors and host health [103–105]. Hansen et al. proved that dietary vitamin B_12_ did not affect transcription of *tcn1* and *tcn2* in the proximal intestine and in the distal intestine, and the *cubn* and *amn* were expressed in the distal intestine but were not affected by diet [106]. Kelly et al. had proved that oral high-dose B_12_ supplements is delivered to the distal gut, where it was available to interact with the microbiota, and they also detected higher concentrations of B_12_ in the fecal contents of the mice supplemented with excess vitamin B_12_ [107]. In the present study, we found that higher concentrations of B_12_ were detected in the hindgut of zebrafish fed with high concentrations of B_12_, which is similar to the previous studies. Also, we did not detect the expression of vitamin B_12_ receptors, but it is important to understand the vitamin B_12_ receptor distribution in the maintenance of zebrafish health. Although this study did not prove the upper limit of saturable uptake of vitamin B_12_ in the zebrafish gut, these results showed that higher concentrations of B_12_ were detected in the hindgut of zebrafish supplemented with high concentrations of B_12_, which is similar to the previous studies [107, 108]. Moreover, the dietary administration of vitamin B_12_ significantly increased gut microbial diversity and altered microbial composition in this study. Additionally, it increased the ratio of Bacteroidetes/Firmicutes, which was closely associated with health of gut [68]. Most of the opinions are that the ratio of Bacteroidetes/Firmicutes was associated with obesity [109, 110]. Bin et al. also found that enterotoxigenic *Escherichia coli* infection caused a significant decrease in the ratio of Bacteroidetes/Firmicutes in the gut of piglets and caused diarrhea [111]. These results indicated that Bacteroidetes/Firmicutes played an important role in host health, which was similar with our data. Meanwhile, vitamin B_12_ supplementation significantly reduce the redox potential in the gut, suggesting that vitamin B_12_ could modulate redox homeostasis. High oxygen environment could provide a more suitable environment for pathogenic bacteria to expand their virulence [112]. Disturbances in the redox balance in the gut could potentiate inflammation, impair barrier function, prevent colonization with a healthy microbiome, and threaten host health. Our findings proved that vitamin B_12_ could prevent disturbances of the redox balance, suggesting that vitamin B_12_ reduced the oxygen environment in the gut and provided a suitable living environment for probiotic bacteria, which in turn may promote microbial interactions. The study from Busti et al. proved that the reduction of the oxygen level in the gut environment contributed to the modulation of gut microbiota favoring the presence of obligate anaerobic [113]. Indeed, the supplementation of B_12_ significantly increased the complexity and stability of the gut ecological network. For example, the number of nodes and links increased due to the supplementation of B_12_, indicating enhanced microbial interactions. The gut microbiome contains thousands of microbes that interact with each other to form complicated networks, and this stability of the gut network is considered important for health of host [82]. Correspondingly, major shifts in microbial community composition are often associated with ill health [82, 114]. In the ecological network, the species were used as nodes and their relationships as links [115], which is essential for characterizing species interactions and dynamics of gut network [116]. We also observed much higher modularity in B_12_ addition group. Modularity is a very important concept in ecological network. It could be originated from specificity of interactions, convergent evolution, and natural selection, and it could be important for system stability [117]. Yang et al. demonstrated that probiotic supplementation led to more module to maintain gut microbiota homeostasis [118]. The higher modularity indicated that the supplementation of B_12_ enhance the stability of gut ecology system. Previous studies have suggested that competition promotes stability in the gut ecosystem and that cooperation creates dependencies that foster instability in microbial communities [82]. Indeed, we observed a higher number of negative interactions in B_12_ addition group, suggesting that the supplementation of B_12_ could improve the stability of the gut ecological network. Overall, the data of the present study indicated that vitamin B_12_ is an important regulator in maintaining the interactive relationships between members of the gut microbiota.

Subsequently, we used the PLS-PM model to explore the relationship among the gut microbiota network, the B_12_ supplementation, and pathogen resistance of zebrafish. Before this, a systematic review of the effects of vitamin B_12_ on gut microbiome was conducted by Guetterman et al., which demonstrated that B_12_ was associated with gut microbiome outcomes, including beta diversity, alpha diversity, relative abundance of bacteria, and functional capacity [41]. However, few studies have been conducted to evaluate the impact of vitamin B_12_ on the pathogen resistance of host. The results of PLS-PM showed that B_12_ had no directed correlation with the pathogen resistance, but had directed correlation with the gut microbiota network. Meanwhile, the gut microbiota network had directed correlation with the pathogen resistance of zebrafish. These suggested that vitamin B_12_ could not directly interact with the host to protect the zebrafish against the pathogen infection but affected pathogen resistance by increasing the stability of the gut microbiota network, which further responded to the importance of vitamin B_12_ as a regulator among microbiota for the host. Moreover, the results of west blot analysis showed that B_12_ did not directly affect gut tight junction proteins, but upregulated them through regulating gut microbes. Tight junction protein expression has been demonstrated to control gut barrier functions, and an increase in tight junction protein levels could prevent or reverse pathogen impacts [119]. Until now, it remains unclear what the underlying molecular mechanisms are on the effects of vitamin B_12_ on the gut barrier. However, it is known that commensal bacteria and probiotics have been shown to promote gut barrier integrity both in vitro and in vivo [120–123]. Previous studies demonstrated that Claudin15, Occludin, and Zo-1 were the key proteins in maintaining the integrity of the gut epithelial barrier [124–126]. In this study, the expression of these proteins (Claudin15, Occludin, and Zo-1) was upregulated in the zebrafish receiving B_12_ supplementation. Meanwhile, we found that the effect of vitamin B_12_ on the expression level of gut tight junction proteins disappeared after the antibiotic treatment. We speculated that the effect of vitamin B_12_ on gut tight junction proteins depended on gut microbes. These data suggested that B_12_-influenced gut microbiota network might alleviate the increased gut permeability caused by pathogen infections. However, at this stage, we did not investigate the mechanisms by which the supplementation of B_12_ enhance the stability of gut microbiota network. Deeper verification tests based on meta transcriptome and metabonomic are necessary, and further research on this issue will contribute to better utilization of vitamin B_12_.

## Conclusions

**Fig. 9:**
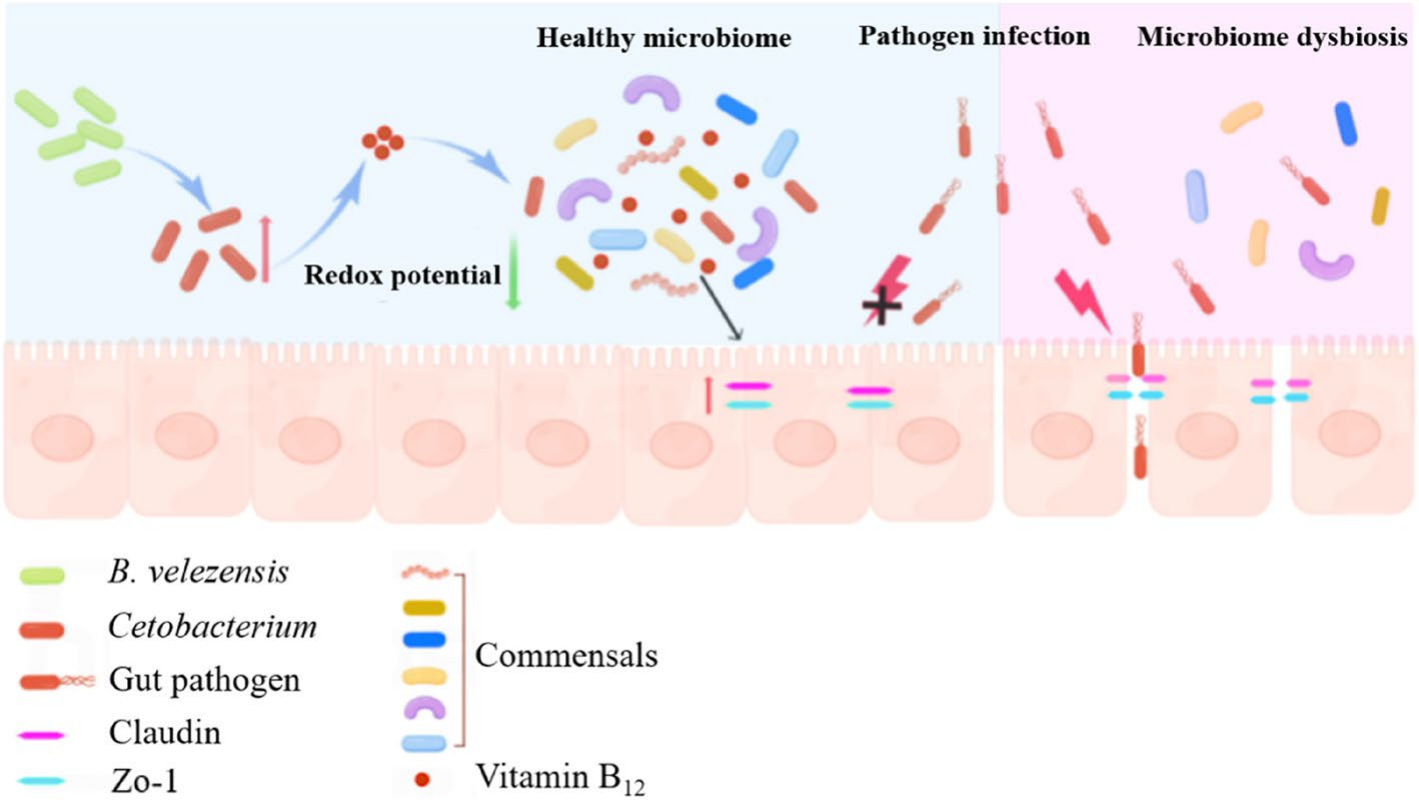
Mechanisms of probiotic protection of the host against pathogen infections. Dietary supplementation with *Bacillus velezensis* BV1704-Y induces an increase in the abundance of the indigenous gut microbiota (*Cetobacterium*) and thus metabolizes sufficient amounts of vitamin B_12_. Vitamin B_12_ is used by the surrounding microbiota to form a more stable and complex gut ecological network while reducing the redox potential in the gut and maintaining the anaerobic state of the intestinal lumen, which further promotes the expression of intestinal tight junction proteins (Claudin15 and Zo-1) and prevents the infestation of *Aeromonas*

The use of probiotics and their metabolites is a practical alternative to promote animal health and prevent disease. We proved that the microbiome alterations influenced by *Bacillus velezensis* BV1704-Y could control the pathogenic load in tissues and improve the survival of zebrafish. Further analysis found that *Cetobacterium*, anaerobic indigenous gut microbe, might be a sensor of health, especially for fish infected with pathogenic bacteria. Genomic analysis and metabolic assays suggested that *Cetobacterium* had the ability to produce vitamin B_12_. Supplementation of vitamin B_12_ reduces the redox potential in the gut, induces alterations in gut microbiome structure and functions, and improves microbial interactions and enhances the stability of the gut microbiota network. Moreover, B_12_ supplementation did not directly influence the pathogen resistance of zebrafish, but by impacting the gut microbiota network. In addition, the stable gut microbiota network upregulated the tight junction proteins of the intestine and protected host against pathogen infections (Fig. 9). Our findings provided a new mechanism of action of probiotics in enhancing host resistance to pathogen infections from the perspective of probiotic action on the gut microbiome. And also demonstrates the novel function of B_12_ as a regulator to enhance microbial interactions. This discovery may deepen the understanding of the impact of probiotics on host health and help to elucidate the health benefits of vitamin B_12_ against pathogen infections.

## Materials and methods

### Zebrafish and experimental design

All experiments were done using 4-month-old AB wild type zebrafish (0.45 ± 0.05 g; 3.5 ± 0.2 cm), which were obtained from the China Zebrafish Resource Center (Wuhan, China). All zebrafish maintained under laboratory conditions with a 14-h light and 10-h dark cycle and adapted to the laboratory conditions for 2 weeks. Each tank containing dechlorinated and aerated water (pH 7.5 ± 0.5) at 28 ± 1 °C. More than 6.5 mg/L of dissolved oxygen was present in the tank.

#### Experiment 1: *B. velezensis* BV1704-Y supplementation experiment

To study the effects of *B. velezensis* BV1704-Y on resistance of zebrafish to *Aeromonas hydrophila* infection, a total of 120 zebrafish were randomly divided into two groups with three 20-L tanks (20 fish/tank) each. Zebrafish were fed with control diet (CK) or *B. velezensis* BV1704-Y-supplemented diet (1 × 10^7^ CFU/g diet) (Y) for 28 days [127]. In order to investigate the role of gut microbiota in the process of probiotic protection of the host against pathogen infection, a common cocktail of antibiotics was used to deplete the intestinal bacteria according to previous studies [49, 50]. Zebrafish received an antibiotic mix consisting of vancomycin (2 g/kg diet), metronidazole (4 g/kg diet), and neomycin sulfate (4 g/kg diet) in the diet for 7 days, and then antibiotics-containing diets were replaced with the control and *B. velezensis* BV1704-Y-supplemented diet (AY), which were fed for 28 days [127], respectively. All diets were 3-mm-diameter pellets. Fish were hand-fed diets at 3% of body weight twice a day (9:00 and 16:00) and consumed all feed offered within 30 s after feeding [106], and the amount of feed intake was about 0.8 g of feed per feeding. The gut, kidney, and liver were collected under aseptic conditions from each zebrafish immediately after the feeding trial, and stored at − 80 °C until further analysis. Then the rest of zebrafish in each group (15 fish in each replicate) were bath infected with *A. hydrophila*, while the control and *B. velezensis* 1704-Y-containing diets were kept fed during the infection period. The experimental design is shown in Fig. 1a.

#### Experiment 2: *C. somerae* CS2105-BJ supplementation experiment

A total of 120 zebrafish were randomly divided into two groups with three 20-L tanks (20 fish/tank) each. Zebrafish were fed with control diet (CK) or *C. somerae* CS2105-BJ-supplemented diet (1 × 10^7^ CFU/g diet) (Ceto) for 28 days, and then received the same treatments as Experiment 1. The experimental design is shown in Fig. 2g.

#### ***Experiment 3: vitamin B***_***12***_*** supplementation experiment***

A total of 120 zebrafish were randomly divided into two groups with three 20-L tanks (20 fish/tank) each. Zebrafish were fed with control diet (CK) or vitamin B_12_-supplemented diet (B) (200 μg/kg diet) for 28 days. Then received the same antibiotic treatment as in Experiment 1 and marked as ACK and AB, respectively. Subsequent operations were the same as in Experiment 1, except that the *B. velezensis* 1704-Y-supplemented diet was replaced with a B_12_-supplemented diet. The experimental design is shown in Fig. 4a.

### Isolation of *Cetobacterium*

Zebrafish from *B. velezensis* BV1704-Y-treated were collected. Gut was removed and grinded into homogenate with 200 μl sterile saline solution. Serial dilutions of 100 μl (10^−3^, 10^−4^, and 10^−5^) containing homogenate were inoculated on BHI agar and incubated at 28 °C for 48 h in an anaerobic environment. The single colony cultured in the medium was chosen and incubated again in the anaerobic bottle with 5 mL BHI broth medium. PCR was utilized with 16S rRNA gene universal primers (27F and 1492R) to verify the bacterial solution. The right samples were kept, and whole-genome sequencing was done for added verification.

### Infection of *Aeromonas hydrophila* AH2006-3 J

*Aeromonas hydrophila* AH2006-3 J (GenBank accession no. OP778940) was isolated in disease outbreak fish pond and screened as the most invasive strain from dozens of different *A. hydrophila* strains. For *Aeromonas hydrophila* infection, zebrafish were immersed in water containing bacteria with a final concentration of 1 × 10^8^ CFU/mL for 10 days [67]. The water and bacteria were changed every 2 days, and mortality was recorded every day. At the end of experiment, the gut, kidney, and liver of zebrafish were sampled under aseptic conditions and stored at − 80 °C until further analysis.

### Bacterial load quantification

Total gut, liver, and kidney DNA was extracted using a QIAamp-DNA Stool Kit (Qiagen, Hilden, Germany). Primers for amplification of genes are listed in Table S1. Amplified sequences were cloned into pMD19-T plasmids (Takara, Dalian, China), to perform a 10-fold dilution and generate a standard curve for calculation of the bacterial load [128, 129]. The values obtained from bacterial copies were calculated relative to the weight of the tissues.

### Microbiome analysis

The gut total DNA was extracted by the QIAamp PowerFecal DNA Kit (Qiagen, Hilden, Germany) according to the manufacturer’s instructions. The V3–V4 regions of the bacterial 16S rRNA gene were amplified using the 341F-806R primers (341F: 5′—CCTAYGGGRBGCASCAG—3′; 806R: 5′- GGACTACNNGGGTATCTAAT—3′) [130]. High-throughput sequencing was performed on the Illumina MiSeq platform at LC-Bio Co., Ltd (Hangzhou, China). In order to create feature tables and feature sequences, the sequences that were obtained from sequencing were subjected to quality filtering and modification. The sequences were aligned using the SILVA Database (v.138) [131] as a reference database, and the taxonomic information was categorized by RDP classifier (v2.2) with 80% confidence [132]. R software (v3.5.2) was used to calculate and display alpha diversity, including the Shannon, Simpson, Richness, and Chao1 indices. Bray–Curtis distance-based PCoA analysis was performed using the vegan R package. Using the LEfSe method [133], we further selected the significant microbiome characteristics in different groups at genus taxonomic rank. PICRUSt2 [134] was used to infer the expected metagenomes and the function of the gut microbiota, and the LEfSe method was used to determine the differentially abundant KEGG pathways between groups.

### Genome sequencing and de novo assembly

The genomic DNA of *Cetobacterium* was extracted for whole-genome sequencing. Following that, Personal Biotechnology Company (Shanghai, China) used the Pacific Biosciences platforms and Illumina MiSeq platforms to sequence the genome. Quality control was performed by using AdapterRemoval [135] and SOAPec [136]. By using SPAdes [137] and A5-miseq [138], the filtered reads were assembled to create scaffolds and contigs. The data received by Pacbio platform sequencing were assembled using Canu software [46]. Subsequently, all assembled results were combined to produce a comprehensive sequence. Finally, using pilon software [139], the genome sequence was obtained after the rectification. The complete genome sequence was deposited at GenBank under the BioProject accession number CP092519-CP092525.

### Determination of vitamin B_12_ and redox potential

To extract vitamin B_12_ from the hindgut, the contents were disrupted by boiling for 15 min in 0.1 M phosphate buffer containing 0.01% potassium cyanide at pH 6.0. After centrifugation at 4500 × *g* for 15 min, the supernatants were collected and passed through 0.22-μm filters [59]. Vitamin B_12_ was detected using a modified HPLC method that was previously reported [59, 140]. At room temperature, all chromatographic separations were carried out. The mobile phases were a mixture of methanol with 0.1% formic acid (A) (Aladdin, Jinan, China) and ultra-purified water with 0.1% formic acid (B), which was degassed by an ultrasonic water bath; the flow rate was 0.5 mL per min. The gradient elution was programmed as follows: 0–2 min, 20% A; 2–3 min, 20–25% A; 3–11 min, 25–35% A; 11–19 min, 35–20% A; 20–22 min, 100–100% A; 22–26 min, 100–20% A; and 26–36 min, 20% A. A Diode Array Detector (1260 Infinity II, US) was used to measure the column eluate at 361 nm, and the injection volume was 100 μl.

For the redox assessment, 20 mg of gut contents was diluted in 2 mL of distilled water and centrifugated at 8000 rpm for 10 min. The redox potential was measured in accordance with the manufacturer’s instructions using a pH/redox meter (REX, Shanghai, China) [73, 74].

### Network analysis

The Molecular Ecological Network Analysis (MENA) pipeline (http://ieg2.ou.edu/MENA/), as previously published [81], was used to conduct network analysis to examine microbial relationships and network complexity of gut microbiota in various groups. Based on the OTU abundances that had been log-transformed, the Pearson coefficient was determined. Prior to network formation, the relevant similarity threshold (*St*) was automatically determined using random matrix theory (RMT) [78]. Following that, all gut microbial networks were created using the same *St* (i.e., 0.88). The network graphs were displayed using the Cytoscape software (3.8.2).

Various indexes, including average degree, average path distance, average clustering coefficient, connectedness, and modularity were used to describe the characterization of individual nodes in the network and the overall topologies or structures of different networks. To test the significance of the constructed empirical MENs, 100 random networks were generated for each empirical network. The means and standard deviations computed from the 100 randomizations for each characteristic were compared to the corresponding empirical MENs [81].

Each node (i.e., OTU) in networks was evaluated for its connectivity using the metrics of within-module connectivity (*Zi*) and among-module connectivity (*Pi*) to find potential keystone taxa that may have an impact on the patterns of gut microbiota assemblage [81]. This can divide all nodes into four categories: *Zi* > 2.5 and *Pi* > 0.62 indicated network hubs; *Zi* > 2.5 and *Pi* ≤ 0.62 indicated module hubs; *Zi* ≤ 2.5 and *Pi* > 0.62 indicated connectors; and *Zi* ≤ 2.5 and *Pi* ≤ 0.62 indicated peripherals. Connectors, module hubs, and network hubs can all be seen of as potential keystone taxa [141].

### Partial least squares path modeling analysis

The PLS-PM was performed using R package of “plsmp” to quantify the effects of different factors (i.e., bacterial diversity reflected by Shannon and Richness; keystone taxa box was reflected by connectors and hubs; network complexity was reflected by degree and connectedness) on the pathogen resistance of zebrafish, which indicated by pathogen load and mortality.

### Western blot analysis

Gut samples were homogenized in RIPA buffer supplemented with 1% of protease and phosphatase inhibitors (Beyotime Biotechnology, Shanghai, China), and protein content was measured with a BCA Protein Assay kit (CWBIO, Suzhou, China). Total proteins (20–50 μg) were resolved using 10% SDS-PAGE gel electrophoresis and transferred to PVDF membrane (0.22 μm pore; Millipore, Billerica, USA). After blocking with 5% (w/v) skim milk at room temperature for 1 h, membranes were then incubated with the primary antibodies at 4 °C overnight (dilutions of respective antibodies are given in Table S5). Next day, membranes were incubated with the HRP-labeled secondary antibody for 40 min at room temperature and the chemiluminescent substrate was used to detect the protein bands. Densitometry analysis of bands was done using ImageJ software.

## Supplementary Information

**Additional file 1: Figure S1.** Identification, hemolytic assay and antibiotics susceptibility of *Bacillus velezensis* strain 1704-Y (BV1704-Y). a The colony characteristics of BV1704-Y in LB agar plate. b Gram stain status and morphological features of BV1704-Y. **c** Phylogenetic tree constructed by the neighbor-joining method based on the *gyrB* gene sequences. d Hemolytic assay for BV1704-Y. e Antibiotics susceptibility testing of BV1704-Y by the paper disc diffusion assay (1-Ofloxacin; 2-Ampicillin; 3-Ceftazidime; 4-Erythromycin; 5-Chloramphenicol; 6-Polymixin B). The detailed information on antibiotics susceptibility of BV1704-Y is listed in Additional file 2: Table S2. **Figure S2.** Dietary supplemented with *Bacillus velezensis* induced the change of gut microbiota composition in zebrafish. Simpson index (a) and number of observed OTUs (b) comparison among the four different groups (Y, CK, TY and TCK). c Relative abundance of the top 6 phyla in the gut samples from the four different groups. (d) The relative abundances of Fusobacteria in the gut sample from Y and CK. (e) The relative abundances of Fusobacteria in the gut sample from TY and TCK. (f) Different bacterial taxa enriched in the TY and TCK zebrafish by LEfSe (LDA score [log 10] > 3.0). (g-r) Relative abundance of selected different taxa. Data are expressed as box plot. *P < 0.05, **P < 0.01 by Mann–Whitney U test with Bonferroni-adjusted *P*-values. (s) Relative abundance of *Cetobacterium* after antibiotics treated. The statistical difference was examined using Kruskal–Wallis H-test followed by Dunn’s multiple comparisons test with Bonferroni-adjusted *P*-values. **Figure S3.** Heat map of Pearson's correlation coefficients between the top 20 genera and infection status (Y:TY). Dark red indicates a stronger positive correlation, dark blue indicates a stronger negative correlation, and white indicates no correlation. Black asterisk (*) means FDR-corrected *P*-value < 0.05. **Figure S4.** Distribution of genes across KEGG functional categories in the genome of *C. somerae* CS2105-BJ. **Figure S5.** *C. somerae* CS2105-BJ produces B_12_ both in vitro and in vivo. **a** The B_12_ production of CS2105-BJ strain in different growth phases. **b** The content of B_12_ in the gut of fish sampled prior to bath infection. * means FDR-corrected *P*-value < 0.05. **c** The liner regression between the content of B_12_ and the abundance of *Cetobacterium* in the gut of fish (CK and Ceto). r and *P* were obtained by Pearson’s correlation analysis. **Figure S6.** a Vitamin B_12_ content in the intestine of zebrafish in different treatment groups. b survival rate of zebrafish in different groups after infection with *Aeromonas hydrophila*. **Figure S7.** The protective effect of B_12_ on zebrafish is concentration-dependent. a The content of B_12_ in the gut of fish sampled prior to bath infection. CK: The zebrafish were fed a basic diet; 50: The zebrafish were fed a basic diet supplemented with vitamin B_12_ (50 μg/kg diet per day); 100: The zebrafish were fed a basic diet supplemented with vitamin B_12_ (100 μg/kg diet per day); 200: The zebrafish were fed a basic diet supplemented with vitamin B_12_ (200 μg/kg diet per day). Significant differences (*P* < 0.05) between different groups are indicated with different lowercase letters above the bars. b Kaplan–Meier graph of the zebrafish survival in different groups after bath infection with A. hydrophila. * indicates significant difference (*P* < 0.05) between different groups. **Figure S8.** Vitamin B_12_ induces alterations in gut microbiota structure. Microbiota alpha diversity was measured by 16S rRNA gene sequence analysis of the gut content samples using Shannon index, Chao1 index, and Observed_otus. Error bars were median with interquartile ranges. * *P*-value < 0.05. **Figure S9.** Vitamin B_12_ induces alterations in gut microbiota structure and function. a Principal coordinate analysis (PCoA) of bray curtis distance was analyzed based on OTU level for microbiota beta diversity (ANOSIM R = 0.7893, *P* = 0.001). b Phylum-level taxonomic distributions of the microbial communities in gut of zebrafish fed with different diets. **c** Liner discriminant analysis effect size (LEfSe) was used to analyze the difference in microbial abundance between TB and TCK group, The LDA value threshold was set at 4.0. d Bacterial community phenotypes of the gut microbiome were predicted using BugBase. Statistical significance was identified by the Wilcoxon test with false discovery rate (FDR)-corrected pairwise *P* values. *, *P* < 0.05. e Functional alterations of the gut microbiome in zebrafish fed with control (TCK) and B_12_-supplementd diet (TB) after infection with *A. hydrophila*. Statistical significance was determined by using LEfSe, with a *P* value of < 0.05 (Wilcoxon test) and a linear discriminant analysis (LDA) score (log_10_) of > 2.2 being considered significant. **Figure S10.** The heatmap shows relative abundance changes for the bacterial genera among the 50 most abundant in any sample. The relative values in the heatmap (after normalization), represented by colors, show the distribution of bacterial species at the genus level among the samples. Red color represents higher abundance, and blue lower abundances. **Figure S11.** B_12_ affects the relative abundance of anaerobic microbiota in gut. Pearson linear correlation between the relative abundance of anaerobic microbiota and vitamin B_12_ quantification of zebrafish fed with control and B_12_*-*supplemented diet in gut. **Figure S12.** B_12_ affects the gut redox potential. a Intestinal redox potential levels. b Pearson linear correlation between Redox potential and vitamin B_12_ quantification of zebrafish fed with control and B_12_*-*supplemented diet in gut. **Figure S13.** B_12_ affects the ecological network of gut microbiome. Demonstration of constructed molecular ecological networks generated using the Molecular Ecological Network Analysis (MENA) pipeline based on OTU relative abundances of gut microbiota. Each link denotes a correlation between two nodes, and each node stands in for a single OTU. Smaller network modules (between 2 and 5 nodes) are depicted in gray, whereas larger network modules (above 5 nodes) are shown in various colors. **Figure S14.** Network analyses for TCK and TB. Large modules (> 5 nodes) are shown in circular layout. Positive and negative correlations are indicated by red and green connections, respectively. The module ID of each large module is indicated by M1 to M11. **Figure S15.** Vitamin B_12_ affect the relative mRNA expression of *Zo-1*, *Occludin*, and *Claudin15*. Relative mRNA expression of *Zo-1*, *Occludin*, and *Claudin15* in different groups. Data were normalized for *β-actin* expression and expressed as fold change. Values represent means ± SD. Significant differences (*P* < 0.05) between different groups are indicated with different lowercase letters above the bars. CK: The zebrafish were fed a basic diet; B: The zebrafish were fed a basic diet supplemented with vitamin B_12_; ACK: The zebrafish treated with antibiotics for 7 days prior to administration of basic diet; AB: The zebrafish treated with antibiotics for 7 days prior to administration of B_12_; TCK: The zebrafish were fed a basic diet and then bath infected with *A. hydrophila*; TB: The zebrafish were fed a basic diet supplemented with vitamin B_12_ and then bath infected with *A. hydrophila*. TACK: The zebrafish treated with antibiotics for 7 days prior to administration of basic diet, and then fed a basic diet and then bath infected with *A. hydrophila*; TAB: The zebrafish treated with antibiotics for 7 days prior to administration of basic diet, and then fed a basic diet supplemented with vitamin B_12_ and then bath infected with *A. hydrophila*. **Figure S16.** *B. velezensis*/*Cetobacterium*/Vitamin B_12_ affect the relative mRNA expression of *Zo-1*, *Occludin*, and *Claudin15*. Relative mRNA expression of *Zo-1*, *Occludin*, and *Claudin15* in different groups. Data were normalized for *β-actin* expression and expressed as fold change. Values represent means ± SD. Significant differences (*P* < 0.05) between different groups are indicated with different lowercase letters above the bars. CK: The zebrafish were fed a basic diet; Y: The zebrafish were fed a basic diet supplemented with *B. velezensis*; B12: The zebrafish were fed a basic diet supplemented with vitamin B_12_; Ceto: The zebrafish were fed a basic diet supplemented with *Cetobacterium*; TCK: The zebrafish were fed a basic diet and then bath infected with *A. hydrophila*; TY: The zebrafish were fed a basic diet supplemented with *B. velezensis* and then bath infected with *A. hydrophila*; TB12: The zebrafish were fed a basic diet supplemented with vitamin B_12_ and then bath infected with *A. hydrophila*; TCeto: The zebrafish were fed a basic diet supplemented with *Cetobacterium* and then bath infected with *A. hydrophila.*

**Additional file 2: Table S1.** Sequences of primers used for qPCR analysis. **Table S2.** Susceptibility of *Bacillus velezensis* 1704-Y to 6 different antibiotics. **Table S3.** Statistics for short-read Illumina sequencing. **Table S4.** The detailed taxonomic information for each node. **Table S5.** List of antibodies used for western blots.

**Additional file 3.**

## Data Availability

The 16S rRNA gene sequencing data and the genomic data generated in this study have been deposited in the National Center for Biotechnology Information (NCBI) Sequence Read Archive under the accession number PRJNA902176, PRJNA907251, and CP092519-CP092525. Additional datasets used and/or analyzed during the current study are available from the author on upon request.
