# Supplementary material for: Vitamin B12 produced by Cetobacterium somerae improves host resistance against pathogen infection through strengthening the interactions within gut microbiota
Source: Microbiome. 2023 Jun 15;11:135. doi: 10.1186/s40168-023-01574-2 (PMC10268390; doi:10.1186/s40168-023-01574-2)
Supplement: Supplementary file 4 — Additional file 3. [file 40168_2023_1574_MOESM3_ESM.docx]

**Additional file 3**

**Supplemented methods**

**Zinc substitution experiment**

A total of 240 zebrafish were randomly divided into four groups with three 20-L tanks (20 fish/tank) each. Zebrafish were fed with control diet (CK), *C.* somerae supplemented diet (Ceto), zinc supplemented diet (Zn) (150 mg/kg diet) [1,2], *Cetobacterium* and excess zinc combined diet (Ceto+Zn) for 28 days. The gut was collected under aseptic conditions from each zebrafish immediately after the feeding trial, and stored at -80^o^C until further analysis. Then the rest of zebrafish in each group (15 fish in each replicate) were bath infected with *A. hydrophila*.

**Isolation of *B. velenzensis***

The gut of healthy zebrafish was isolated under sterile conditions, rinsed with sterile Phosphate Buffer Saline (PBS). The gut was homogenized in 1 mL sterile PBS and the suspension was spread on Luria Bertani (LB) agar plates after 100 folds dilution with sterile PBS. The plates were incubated at 28 ^o^C for 24 h [3]. The single colony cultured in the medium was chosen and incubated again in the bottle with 5 mL LB broth medium. The genomic DNA of the isolated strain was extracted by Genomic DNA Kit (Tiangen Biotech, China). The 16S rDNA and sequence of gyrB were amplified from the genomic DNA. The PCR products were directly sent to sequencing (Shenggong Biotechnological Ltd., Shanghai, China), and the obtained nucleotide sequence was used for a NCBI BLAST to perform the similarity analysis and the multi-sequencing alignment.

**Hemolytic assays**

The hemolysis test was used to detect the production of hemolysin in order to exclude the potential pathogenicity. The suspension was streaked on plates containing goat blood-based agar and incubated at 28 °C for 24 h. The hemolytic activity was determined classified by the appearance of the hemolysis. α-hemolysis showed a partial and green circle around the bacterial colonies; β-hemolysis showed a clean hemolysis zone around colonies; γ-hemolysis showed no change on the agar plates around the colonies [4].

***B. velenzensis* 1704-Y athogenicity assay**

*B. velenzensis* 1704-Y were inoculated in LB with an incubator shaker at 28 °C for 12 h. The cells were collected by centrifugation at 5000×g for 10 min and washed twice with sterilized PBS, and then the pellet was subjected to 10-fold serial dilutions with PBS. The zebrafish were intraperitoneally injected with 0.1 mL of strain suspension at concentrations of 10^6^, 10^7^, 10^8^, 10^9^, 10^10^ CFU /mL, respectively. Meanwhile, the grass carp in control group was intraperitoneally injected with 0.1 mL of PBS. Each group included 15 zebrafish (0.45 ± 0.05 g) and performed in three replicates. During the experiment, the activity and behavior of each fish were observed, and the mortalities were recorded daily for 7 days.

**Antibiotic sensitivity assay**

The susceptibility of *B. velenzensis* 1704-Y to antibiotics was assessed by the disc diffusion method [5,6]. A volume of 100 μL of *B. velenzensis* 1704-Y with the cell density of 10^8^ cfu/mL was spread on LB agar plates, and then the antibiotic discs containing vancomycin, gentamicin, kanamycin, tetracycline, ampicillin and streptomycin were respectively attached on the surface of LB agar and incubated at 28 °C for 24 h [7,8]. According to the inhibition-zone diameters, antibiotic sensitivity was expressed in terms of susceptible (S), resistance (R) or intermediate resistant (I).

**References**

[1] Roberto VP, Martins G, Pereira A, Rodrigues S, Grenha A, Pinto W, et al. Insights from dietary supplementation with zinc and strontium on the skeleton of zebrafish, Danio rerio (Hamilton, 1822) larvae: From morphological analysis to osteogenic markers. J Appl Ichthyol. 2018;34(2):512-523; doi: 10.1111/jai.13664.

[2] Wang, Zhu, Guo, Zhang, Jia. Influence of Different Dietary Levels of Zinc on Performance, Vitamin B12, and Blood Parameters in Lambs. Int J Vitam Nutr Res. 2006;76(6):353-358; doi: 10.1024/0300-9831.76.6.353.

[3] Li J, Wu Z-B, Zhang Z, Zha J-W, Qu S-Y, Qi X-Z, et al. Effects of potential probiotic Bacillus velezensis K2 on growth, immunity and resistance to Vibrio harveyi infection of hybrid grouper (Epinephelus lanceolatus♂ × E. fuscoguttatus♀). Fish Shellfish Immunol. 2019;93:1047-1055; doi: 10.1016/j.fsi.2019.08.047.

[4] Luis-Villaseñor IE, Macías-Rodríguez ME, Gómez-Gil B, Ascencio-Valle F, Campa-Córdova ÁI. Beneficial effects of four Bacillus strains on the larval cultivation of Litopenaeus vannamei. Aquaculture. 2011;321(1):136-144; doi: 10.1016/j.aquaculture.2011.08.036.

[5] National Committee for Clinical Laboratory Standards (NCCLS). Performance Standards for Antimicrobial Susceptibility Testing; Ninth Informational Supplement. Wayne, Pensilvania document M100-S9, 1999, Vol.19. No.1, Table 2I. https://doi.org/10. 1016/S0196-4399(01)88009-0.

[6] Rojo-Bezares B, Sáenz Y, Poeta P, Zarazaga M, Ruiz-Larrea F, Torres C. Assessment of antibiotic susceptibility within lactic acid bacteria strains isolated from wine. Int J Food Microbiol. 2006;111(3):234-240; doi: 10.1016/j.ijfoodmicro.2006.06.007.

[7] Additives EPo, Products or Substances used in Animal F. Guidance on the assessment of bacterial susceptibility to antimicrobials of human and veterinary importance. EFSA Journal. 2012;10(6):2740; doi: 10.2903/j.efsa.2012.2740.

[8] Sharma P, Tomar SK, Sangwan V, Goswami P, Singh R. Antibiotic Resistance of Lactobacillus sp. Isolated from Commercial Probiotic Preparations. Journal of Food Safety. 2016;36(1):38-51; doi: 10.1111/jfs.12211.
